# Modeling Long-Term Graft Survival With Time-Varying Covariate Effects: An Application to a Single Kidney Transplant Centre in Johannesburg, South Africa

**DOI:** 10.3389/fpubh.2019.00201

**Published:** 2019-07-25

**Authors:** Okechinyere J. Achilonu, June Fabian, Eustasius Musenge

**Affiliations:** ^1^Division of Biostatistics and Epidemiology, School of Public Health, Faculty of Health Sciences, University of the Witwatersrand, Johannesburg, South Africa; ^2^Wits Donald Gordon Medical Centre, Faculty of Health Sciences, University of the Witwatersrand, Johannesburg, South Africa

**Keywords:** graft survival, time varying covariate effect, Cox PH model, purposeful selection, additive hazard models

## Abstract

**Objectives:** Patients' characteristics that could influence graft survival may also exhibit non-constant effects over time; therefore, violating the important assumption of the Cox proportional hazard (PH) model. We describe the effects of covariates on the hazard of graft failure in the presence of long follow-ups.

**Study Design and Settings:** We studied 915 adult patients that received kidney transplant between 1984 and 2000, using Cox PH, a variation of the Aalen additive hazard and Accelerated failure time (AFT) models. Selection of important predictors was based on the purposeful method of variable selection.

**Results:** Out of 915 patients under study, 43% had graft failure by the end of the study. The graft survival rate is 81, 66, and 50% at 1, 5, and 10 years, respectively. Our models indicate that donor type, recipient age, donor-recipient gender match, delayed graft function, diabetes and recipient ethnicity are significant predictors of graft survival. However, only the recipient age and donor-recipient gender match exhibit constant effects in the models.

**Conclusion:** Conclusion made about predictors of graft survival in the Cox PH model without adequate assessment of the model fit could over-estimate significant effects. The additive hazard and AFT models offer more flexibility in understanding covariates with non-constant effects on graft survival. Our results suggest that the period of follow-up in this study is long to support the proportionality assumption. Modeling graft survival at different time points may restrain the possibility of important covariates showing time-variant effects in the Cox PH model.

## 1. Introduction

The incidence and prevalence of end-stage kidney disease (ESKD) have significantly increased in developing countries, such as South Africa (1). Patients with ESKD have an increased risk of premature death on chronic dialysis therapy and for long term survival, kidney transplantation is the treatment of choice (2). A successful kidney transplant increases the life-expectancy and quality of life of a patient with ESKD. Despite advances in the use of immunosuppressants, recipient and donor factors still compromise the efficacy of a kidney transplant outcome, especially for long-term survival (3, 4). This has brought increased interest in identifying these factors using statistical methods, such as survival analysis. In kidney transplant studies, time-to-graft failure or patient death is usually the event of interest.

Beyond the Kaplan-Meier (KM) estimator, most kidney transplant studies employ the Cox Proportional hazard (PH) model to analyse whether individual patients or donor's characteristics influence the probability of Graft survival (GS) or graft failure (GF). The framework of proportional hazard assumption under the Cox PH model states that factors under study act multiplicatively on the baseline hazard function and either increase or decrease the baseline function at a constant rate (5). This fundamental assumption may not be tenable in kidney transplant studies because the effect of recipient age may impose a strong effect immediately after kidney transplant but gradually fades with time. In this situation, a hazard ratio (HR) does not suggest the same magnitude or size on the survival time. Therefore, the variable is said to have a time-varying effect on survival. Assessing the PH assumption should be the fundamental aspect in the use of the Cox PH model because violation of this assumption could lead to misleading of the resulting parameter interpretation (6). However, if the assumption of PH is violated for any covariate, a more flexible model which does not condition on constant proportional could offer more insight about the relationship between graft survival and the risk factors.

One of these models is the Aalen's additive hazard model (7), which specifies how the covariates impact additively on the hazard, but the effects of the covariates are allowed to vary freely over time. As, however, the closest version of an additive hazard model which is analog to the Cox hazard model is the Lin and Ying (8) model. It assumes the covariates act additively upon an unknown baseline hazard and their effects are constant. Conversely, the effects of the covariates in the model may be constant or time-varying. McKeague and Sasieni (9) proposed a version of the additive model that accommodates both constant and time-varying covariates effects. Although several authors advocated and used the additive hazard models for survival time data, however, the additive hazard model is rarely used in survival data analysis, more especially in kidney transplant research due to lack of familiarities with the model (10, 11). Similarly, the parametric accelerated failure time (AFT) models accommodate time-varying covariates effects. The effect of covariates in an AFT model is constant and act multiplicatively on the survival times (12), and the covariates accelerate or decelerate the occurrence of events of interest i.e., a predictor effect acting to either accelerate or decelerate graft survival time. The formulation of these models allows the estimation of a time ratio (TR) and the regression coefficients are estimated with the method of full maximum likelihood. Parametric survival models were considered by Hashemian et al. (13), in analyzing survival after kidney transplant and noted that parametric survival models provide a more suitable description of the survival data compared with the Cox PH model.

This study is motivated by previous studies on the statistical analysis of kidney transplants done in South Africa (14–16). These studies focused on the comparison of patients and GS or identification of factors that influence survival using the KM estimator and standard Cox PH model. As an extension to these previous studies, this study aims to use a more rational and methodical approach to (i) identify factors that influence long-term GS using purposeful model building strategy, (ii) affirm the importance of assessing the PH assumption in Cox PH model, and finally (iii) show the need to consider additive hazard and AFT models as a complement to the Cox model when the PH assumption is not tenable.

## 2. Patients and Method

We studied patients ≥18 years that underwent their first kidney transplant at Charlotte Maxeke Johannesburg Academic Hospital between 1984 and 2000. This is a retrospective cohort study, which involves 915 adult patients. Patients were followed-up after transplant, and information detailing patients, donors and transplant characteristics were recorded. GS was defined as the period from transplant to GF, loss to follow-up or end of the study. That is patients were right-censored if the graft did not fail by the end of the study or the patients were lost to follow-up (graft failure: 1, censored or alive: 0). Deaths with functioning grafts were not captured in this study. GF rates were computed as the ratio of the number of failed grafts to patient-years (PY) of follow-up and expressed as failure rates per 1,000 PY. Predictor variables or covariates for inclusion in this study were identified from literature using factors shown to significantly influence graft survival (16–18).

**Figure 1:**
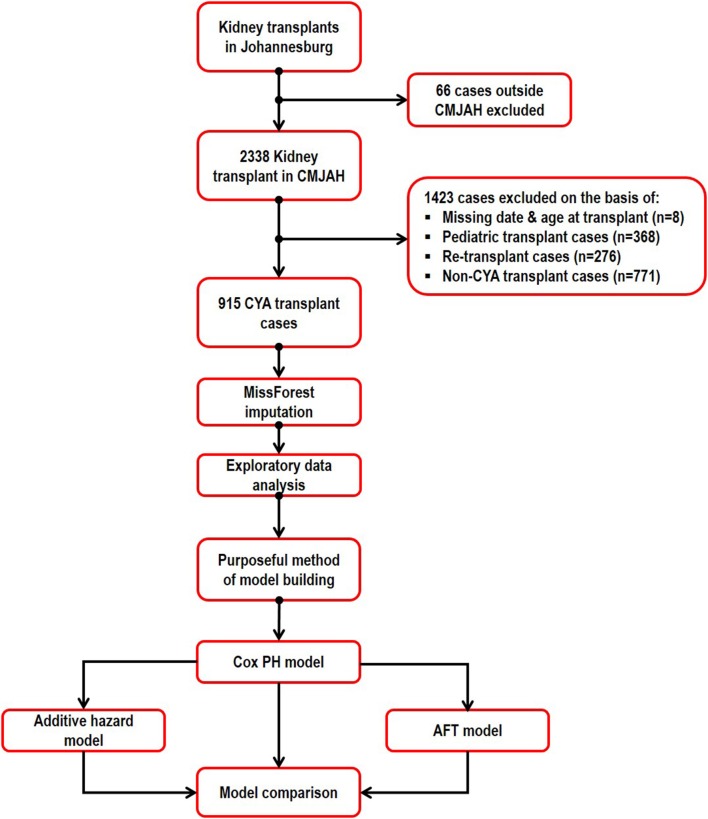
Flowchart of data extraction and study design.

The covariates considered in this study are not time-dependent because they were only measured at the beginning of the study. There is no relationship between each variable missingness and the values of the variable or other variables in the study. Nonetheless, we numerically verified the assumption of missing completely at random using the Little's test of MCAR (19). MissForest based imputation method (20) was used to replace the missing data with reasonable values. MissForest is a non-parametric imputation method that can simultaneously impute different types of variables and its algorithm is based on random forest. There is no need to specify the tweaking parameter or the distribution of the data in the algorithm. For each variable with missing observation, the algorithm fits a random forest model using the rest of the variables in the dataset and then predict the missing values for that variable. The imputation procedure continuously run interactively and performance between iterations are assessed until a stopping criterion is reached. This is done in a repeated approach for all the variables with missing value in the dataset. For the continuous variable (donor age) with missing value, we assessed the performance of the imputation algorithm using the normalized root mean square error and for the categorical variables with missing, we used the proportion of falsely classified entries (21). The data were summarized and relevant information available for the patients were extracted. The analysis steps are described in Figure 1.

## 3. Survival Analysis Methods

Let *T*_*i*_ be a random variable that represents GF time for patient *i* with characteristics *X*_*i*_, a p-dimensional covariate vector. Suppose *C*_*i*_ denotes right censoring times, the distribution of *C*_*i*_ is independent of *T*_*i*_ such that *min*(*T*_*i*_, *C*_*i*_) is observed. Typically, a survival dataset $D_{m}$ consists of *m* i.i.d. representative observations (*T*_*i*_, δ_*i*_, *X*_*i*_), *i* = 1, …, *n* and δ_*i*_ = *I*[*T*_*i*_ ≤ *C*_*i*_, δ = 1 or *T*_*i*_ > *C*_*i*_, δ = 0] is defined as censoring indicator.

### 3.1. Cox Proportional Hazard Model

The Cox PH model was used to analyse the effect of the study predictors on GS. The purposeful method of variable selection employed in this study was based on the Cox PH model (5). First, the effects of all the study covariates on graft survival were assessed univariately with the Cox PH model (22). If *T*_*i*_ follows the Cox PH model, then the hazard function for *T*_*i*_ at time *t* > 0 conditional on *X*_*i*_ is given by

(1) $$\begin{array}{r} h\left(t|X_{i}\right)=h_{0}\left(t\right)\exp\left(X_{i}^{\prime }\beta \right), \end{array}$$

where *h*_0_(*t*) is arbitrary, the unspecified non-negative function of time known as baseline hazard. It corresponds to the hazard when all predictor variables are equal to zero. β denotes the vector of the regression coefficients, which is estimated using the partial likelihood method. The term exp(*X*′β) depends on covariates, but not time. Significant variables at a 25% level of significance in the univariable analysis were included in the multivariable Cox PH model as applied by Hosmer et al. (5) and Bursac et al. (23). Variables were excluded from the model sequentially if they were neither significant predictor of graft survival nor confounders. The procedure for the purposeful method of variable selection is detailed in Hosmer and Lemeshow (5). Under the Cox PH model, a continuous covariate is assumed to have a log-linear functional form. Sometimes the effect of a covariate may not be in a linear association with the log-hazard. Hence, assuming a linear effect when a non-linear effect is applicable results in misspecification, which definitely affects the estimated coefficients and standard errors. The functional form of the continuous covariates was assessed using the plot of martingale residual from a null model and cumulative sums of martingale residual plot (24–26).

One restrictive assumption of the Cox PH model is the PH assumption. The hazard of two individuals with covariates *X*_1_ and *X*_2_ is said to be proportional when the hazard ratio is constant over time. That is, $HR=\frac{h\left(t,X_{1}\right)}{h\left(t,X_{2}\right)}=\frac{h_{0}\left(t\right)\exp\left(\beta X_{1}\right)}{h_{0}\left(t\right)\exp\left(\beta X_{2}\right)}=\frac{\exp\left(\beta X_{1}\right)}{\exp\left(\beta X_{2}\right)}=\exp\left\{\beta \left(X_{1}-X_{2}\right)\right\}$. This implies that the ratio of the two hazards remains proportional or constant over time. When the hazard ratio (HR) of a variable is not constant over time, the covariate is said to have a non-proportional or time-varying effect on survival, which suggests that the effect of the covariate changes over time. Test and graphical methods based on scaled Schoenfeld residuals (${\text{r}}_{i}^{w}=n_{e}\text{var}\left(\hat{\beta }\right){\text{r}}_{i}$) and technique based on cumulative sums of martingale residuals ($U\left(\hat{\beta },t\right)=\overset{n}{\underset{i=1}{\sum }}X_{i}{\hat{M}}_{i}\left(t\right)$) (25, 27) were used to verify the validity of proportionality for each selected covariate in the final model. The scaled Schoenfeld residuals vs. time were plotted for each covariate. Under common definition, these residuals are expected to randomly distribute around the zero line slope if proportionality is valid. Also, the observed processes plotted along with 50 simulated processes under the null hypothesis of no model misspecification were compared. The non-proportional hazard assumption for any covariate is revealed if the observed processes are atypical of the simulated processes. A clear lack of fit could be concluded for the Cox PH model due to time-varying covariates effects in the model, which violates the PH assumption.

### 3.2. Additive Hazard Model

To circumvent PH assumption and characterize the nature of the time-varying covariates effects through the cumulative regression function plots, we employed the Aalen additive hazard model, given by

(2) $$\begin{array}{r} h\left(t|X_{i}\right)=h_{0}\left(t\right)+X^{\prime }\gamma \left(t\right). \end{array}$$

Similar to model 1, *h*_0_ and γ represent the baseline hazard function and vector of time-varying regression coefficients, which may change in magnitude and even sign over time. The flexibility of the Aalen additive hazard is tempered due to the difficulty indirect estimation of the coefficients function. Hence, the cumulative regression coefficients version is estimated based on the least square estimation of the integrated coefficients $\beta_{i}\left(t\right)=\int_{0}^{t}b_{i}\left(u\right)du,i=1,\ldots ,p$. These effects are graphed against time to investigate if the covariates in model 1 have time-varying or constant effects over time. The more beta is from 0, the higher the impact the coefficients has had on the hazard of graft failure over the period of follow-up. As well, a positive and a negative slope with an increase in covariates indicate an increase and a decrease in hazard, respectively. For a covariate with significant effect, the confidence bands are likely not to include the zero line. Both the Kolmogorov-Smirnov and Cramer Von Mises tests (28) were used to assess the time-invariant effects of the covariates. The cumulative martingale residual was used to assess the fit of the covariates in the Aalen additive hazard model. To further assess the nature of these covariates, we fitted a variation of model 2, given by

(3) $$\begin{array}{r} h\left(t|X_{i}\right)=h_{0}\left(t\right)+X_{a}^{\prime }\gamma_{a}\left(t\right)+X_{b}^{\prime }\gamma_{b}. \end{array}$$

In this version of the additive model, γ_*b*_(*t*) and γ_*a*_ represent a vector of covariates with time-varying and constant effects, respectively. A successive test was done to compare the result of this model and that of the previous models.

### 3.3. Accelerated Failure Time Models

All the significant variables from the Cox PH model were also used to fit AFT models. We used the shape of the hazard function to select the appropriate AFT models, as reported by Khanal et al. (29). The baseline hazard function profile (Figure S1) displays a monotone decreasing hazard, which is closer to log-logistic (when *k* ≤ 1), log-normal (when σ> 1) and Weibull distributions (when γ < 1) (12). The survival functions of the selected distributions are $S\left(t\right)=1-\Phi \left(\frac{\log t-\mu }{\sigma }\right)$, *S*(*t*) = {1+^*e*^^θ^*t*^*k*^}^−1^ and *S*(*t*) = exp(−λ*t*^γ^). The distributions are characterized by the location or scale (μ, θ, λ) and shape (σ, *k*, γ) parameters. In the AFT model, the effect of covariates is constant and act multiplicatively on survival times. The log-linear relationship between the variables and the log of survival time is given by

(4) $$\begin{array}{r} \log T=\mu +\alpha^{\prime }X+\sigma \epsilon , \end{array}$$

where μ is the model intercept, α is a vector of regression coefficients quantitatively expressing the impact of each explanatory variable on the survival time. A negative value of α indicates that survival time increases with decreasing value of the explanatory variable and vice versa. The exp(α′*X*) is usually referred to as the acceleration factor. σ is the scale parameter and ϵ is the error term, which is assumed to have a specific distribution, such as a logistics or normal distribution. The deviation of log*T* from linearity is modeled by the error term. The distribution *T* is based on the probability distribution of ϵ, and the survival function for *T* can be obtained from the survival function of the distribution of ϵ. The Akaike information criterion defined by *AIC* = −2*l* + 2*k* (30), where l is the log-likelihood of the model and k is the total number of parameters in the model, was used to compare the fit of the AFT models. The best performing model was used to compare the results of the Cox PH and Additive hazard models. To draw valid inferences from the best-performing models, Deviance residuals were used to assess the adequacy of the selected model (5). The deviance residual is express as

(5) $$\begin{array}{r} r_{Di}=\text{sign}\left(r_{Mi}\right){\left[-2\left\{r_{Mi}+\delta_{i}\log\left(\delta_{i}-r_{Mi}\right)\right\}\right]}^{1/2}, \end{array}$$

where the quantity *r*_*Mi*_ is the martingale residual. The sign function defined by sign(.) takes the value −1 or +1 if its argument is negative or positive, respectively. The deviance residuals are normalized transformations of the martingale residuals and have a mean of zero. If the model is valid, the *r*_*Di*_ are more symmetrically distributed around zero compared to *r*_*Mi*_. The R codes used to perform the analysis is included in the Supplementary Material.

## 4. Results

### 4.1. Descriptive Statistics

**Figure 2:**
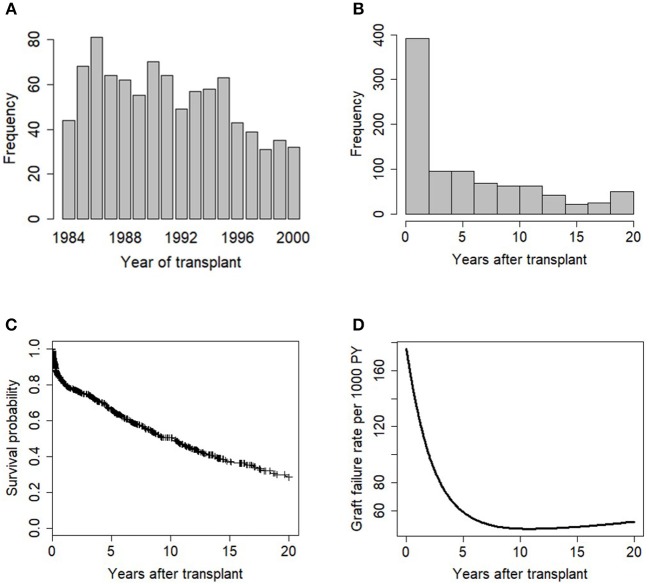
Exploratory data analysis of the transplant data showing **(A)** barplot of years of kidney transplantation, **(B)** histogram plot of graft survival time variable, **(C)** KM plot of graft survival, (+) indicates censoring, and **(D)** Smoothed graft failure rate per 1,000 PY.

The descriptive information available for the 915 patients in this study is summarized in Table 1. Majority of the patients (85%) received a kidney from cadaveric donors, and white patients accounted for 56% of the total patients in the study. The unadjusted graft failure rates for the study variable categories are also listed in Table 1. Most transplant cases concentrated before 1992, which is the midpoint of kidney transplantation in this study (Figure 2A). We observed 43% cases of graft failure by the end of the study; hence, the censoring rate is about 58%. Graft survival at 1, 5, 10 and 15 years are 81% (95% CI: 78–84%), 66% (95% CI: 63–70%), 50% (95% CI: 47–55%), and 37% (95% CI: 32–42%), respectively. The median follow-up was ~10 years, about 17% grafts failed after the 1st year of follow-up and this period has the highest hazard rate of GF (Figures 2B–D). 18% of the cases have missing observations in their records and there are no missing values in the time variables. The Little's MCAR results show that these observations are missing completely at random (*p*-value = 0.206). MissForest method of imputation was used to address the issue of missing data in this study and the reliability of the method was assessed. The out-of-bag errors estimated by missForest for the continuous variable and categorical variables are 0.02 and 0.14, respectively. This shows good performance of missForest in imputing missing data because the values are close to zero than 1.

**Table 1:** Characteristics of kidney transplant recipient in CMJAH from 1984 to 2000 and partial likelihood ratio test *p*-value for all the study covariates.

**Variable** **X code**		**Mean (range)** **/ *n* (%)**	**Event**	**Rate per 1,000** **(95%CI)**	***p*-value**
X1	Recipient age	38.0 (18–68)			<0.001
X2	Donor age	28.4 (1–65)			0.100
					
X3	Clinical acute rejection				
	No	363 (39.7)	151	71.9 (61.5–84.1)	0.400
	Yes	552 (60.3)	233	79.5 (69.9–90.4)	
X4	Histological acute rejection				
	No	772 (84.4)	337	74.0 (66.6–82.3)	0.200
	Yes	143 (15.6)	47	98.5 (74.0–131.1)	
X5	Donor type				
	Cadaveric	781 (85.4)	351	83.2 (75.0–92.4)	<0.001
	Living	134 (14.6)	33	40.7 (29.1–56.9)	
X6	Recipient ethnicity				
	White	517 (56.5)	196	63.0 (54.8–72.4)	<0.001
	Non-white	398 (43.5)	188	95.9 (83.2–110.6)	
X7	Diabetes at transplant				
	No	854 (93.3)	348	74.4 (66.9–82.6)	0.200
	Yes	61 (6.7)	36	94.6 (93.3–130.6)	
X8	Donor-recipient gender				
	m-m	377 (41.2)	155	71.6 (61.0–84.0)	0.600
	f-f	120 (13.1)	56	85.0 (64.8–111.6)	
	f-m	243 (26.6)	107	81.7 (67.6–98.6)	
	m-f	175 (19.1)	66	77.8 (61.0–99.2)	
X9	Donor-recipient blood group				
	Mismatched	91 (9.9)	31	63.2 (44.4–89.8)	0.300
	Matched	824 (90.1)	353	77.7 (69.9–86.2)	
X10	Delayed graft function				
	No	582 (63.6)	248	66.2 (58.5–74.9)	<0.001
	Yes	333 (36.4)	136	106.2 (89.7–125.7)	
X11	Renal ESKD				
	No	519 (56.7)	235	81.7 (71.9–92.8)	0.100
	Yes	396 (43.3)	149	69.3 (59.1–81.1)	
X12	Hypertension ESKD				
	No	628 (68.6)	252	69.4 (61.4–78.4)	0.020
	Yes	287 (31.4)	132	94.7 (79.9–112.3)	
X13	Urological ESKD				
	No	846 (92.5)	361	78.0 (70.5–86.5)	0.200
	Yes	69 (7.5)	23	56.1 (37.3–84.4)	
X14	Inherited ESKD				
	No	828 (90.5)	351	79.3 (71.5–87.9)	0.060
	Yes	87 (9.5)	33	54.5 (38.9–76.3)	
X15	Surgical complication				
	No	599 (65.5)	254	67.0 (59.1–76.1)	0.040
	Yes	316 (34.5)	130	90.3 (75.9–107.4)	

### 4.2. Result From the Cox Proportional Hazard Model

The first step considered in the model building procedure was to explore the relationship between each covariate and graft survival time, univariately. At 25% level of significance, evidence of association with GS is suggested for some variables (Table 1). These variables were deemed candidate for inclusion in the multivariable model.

The multivariable model containing all the significant covariates in the univariable analysis was fitted (Table S1). In order to simplify the model, *p*-values of the covariates based on the partial likelihood test were examined. “Donor age” has the largest *p*-value (*p* = 0.654), which is not statistically significant. Omitting this covariate and refitting the model results in the likelihood ratio (LR) test of 0.202 (Table 2). This is not significant (*p* = 0.653) at 5% level, indicating no improvement over the full model. Furthermore, the change in coefficients $\left(\Delta \hat{\beta }\right)$ for each covariate remaining in the model was compared with the original model, the result (Table 2) shows that donor age is neither a significant predictor of graft survival nor a confounder. Next, “Histological acute rejection” and “Urological ESKD” were subsequently removed from the model. The LR tests with *p*-values of 0.349 and 0.227, respectively, show that the model without these covariates is not statistically different from the model with these covariates (Table 2). However, the removal of “Urological ESKD” influenced the coefficients of “Renal disease ESKD” and “Hypertension” by more than 15%. “Urological ESKD” would have been retained in the model if “Renal disease ESKD” and “Hypertension” were significant predictors of graft survival at 10% level of significance. Therefore, we considered “Urological ESKD” as an unimportant confounder and exclude the three variables from the model.

**Table 2:** Partial likelihood ratio test indicating the effect of deleting covariates that are not significant in the multivariable analysis and their highest impact in coefficient change for other covariates.

**Model**	**$-2LL\left(\hat{\beta }\right)$**	**$\Delta \left(-2LL\left(\hat{\beta }\right)\right)$**	**df**	***p*-value**	**%$\Delta (\hat{\beta })$^a^**
Model 1	4,538.881				
Model 1 − X2	4,539.082	0.202	1	0.653	5.08
(Model 1 − X2) − X4	4,539.961	0.878		0.349	5.67
((Model 1 − X2) − X4) − X13)	4,541.420	1.461	1	0.227	31.91
((Model 1 − X2) − X4) − X13) − 4 (X11 + X12) = Model 2	4,542.927				
Model 2 − X14	4,544.815	1.888	1	0.169	7.31
(Model 2 − X14) − X15 = Model 3	4,544.815	2.122	1	0.145	4.33
Model 3 + X8 = Model 4	4,539.907	4.908	3	0.027	5.50
Model 4 + X9	4,539.907	<0.001	1	0.997	0.06
Model 4 + X3	4,539.467	0.440	1	0.507	7.00

a *Highest change observed in covariates coefficients after deleting each covariate*.

There is no significant change in the value of $-2LL\left(\hat{\beta }\right)$ on deleting “inherited ESKD” and “Hypertension” from model 2, sequentially (*p*-value = 0.169 and 0.145). The deletion did not confound the relationship of any covariate remaining in the model and graft survival. The final covariates in the multivariate model at this stage is shown in Model 3 (Table S1).

In the next stage, “Donor recipient-gender match,” “Donor-recipient blood group match,” and “Clinical acute rejection” that were not significant in the univariable analysis were sequentially added in the multivariable Model 2). Only “Donor-recipient gender” shows a significant relationship with graft survival, with LR test of 4.908 (*p* =< 0.027). Hence, we re-consider this variable at this stage of model building (Model 4; Table 2). The summary of Model 4 is shown in Table S1. We compared the variables selected in the final model with an automated method of variable selection, such as stepwise and best-subset (Table S3). We observed that automated methods are susceptible to selecting more variables, which are not significantly related to GS at 5–10% significant levels.

#### 4.2.1. Assessment of Linearity Assumption

**Figure 3:**
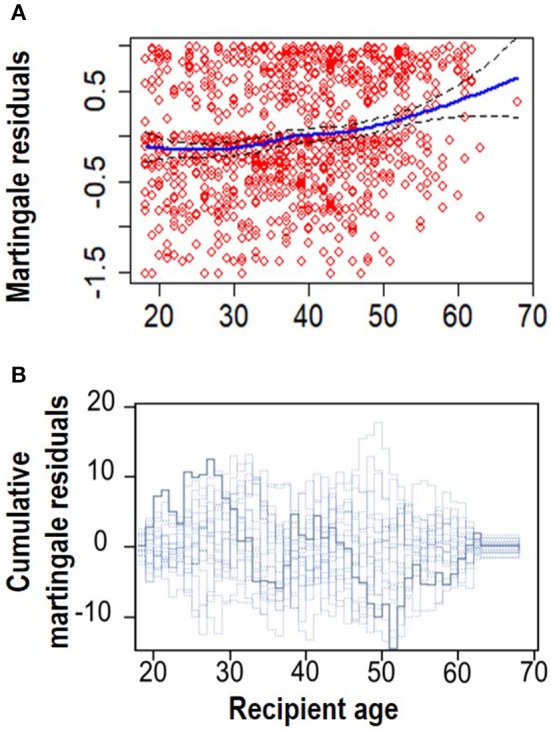
Linearity assumption assessment. **(A)** Smoothed martingale residual plot from a null Cox PH model vs. recipient age. **(B)** Cumulative martingale residuals plot vs. recipient age (*p* = 0.095).

The next step was to assess the functional form of “Recipient age,” as the only continuous variable in the final model (Model 4, Table S1). Figure 3 shows a plot of the martingale residual from a null model and the cumulative martingale residual. The smoothing spline fit shows evidence of linearity for this variable. It is also obvious that the observed processes for this variable are more typical with the 20 simulated realizations from the null distribution with a complimentary *p*-value of 0.100. This indicates that a linear term is needed for “Recipient age” in the model.

#### 4.2.2. Assessment of PH Assumption and Overall Goodness-of-Fit

There was no significant two-way interaction between the covariates in the model at 5% level of significance. We assessed the assumption of the Cox PH model to confirm if the covariates interpreted above only shift the baseline hazard up or down, but does not change over the lifetime of a graft. Figure S2 shows evidence of time-varying effects for some covariates in the model, given that the curves seem not to drift apart steadily, as should be expected in the case of constant effects. Table 3 shows the *p*-values of tests based on the scaled Schoenfeld and cumulative residuals for non-proportional hazard assessment. The results of the two tests support evidence of deviation from the proportionality assumption as shown in Table 3. The results are graphically illustrated for each covariate in the Cox PH model (Figures S3, S4). These figures suggest non-constant effects over time for the aforementioned variables. However, when these covariates interacted with time in the extended Cox PH model, only recipient ethnicity shows a non-constant effect (Table S2). The non-constant effect of these covariates indicate a lack of fit in the Cox PH model, which could lead to misleading parameter interpretation.

**Table 3:** Non-proportionality test in the Cox PH model, *p*-values for scaled Schoenfeld residuals and cumulative residuals (*) tests.

**Variable**	**Rho**	**Chisq**	***p*-value**	*p*-value*
Donor type	−0.061	1.398	0.237	0.100
Delayed graft function	−0.136	7.183	**0.007**	**<0.001**
Diabetes at transplant	0.124	5.870	**0.015**	**0.001**
Recipient ethnicity	−0.067	1.741	0.187	**0.005**
Recipient age	0.071	2.103	0.147	0.320
Donor-recipient gender (f-f)	−0.080	2.437	0.118	0.060
(f-m)	−0.015	0.083	0.773	0.240
(m-f)	−0.018	0.122	0.727	0.900
GLOBAL	NA	25.292	0.001	

*Bold variables represent violation of the PH assumption*.

### 4.3. Result From Additive Hazard Models

**Figure 4:**
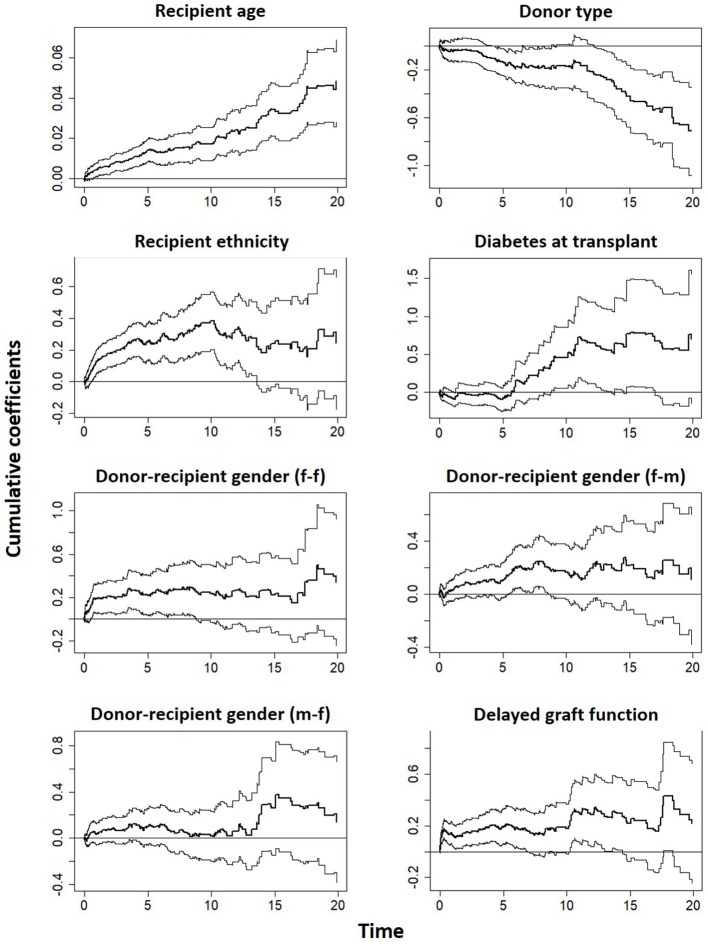
Estimates of cumulative hazard risk with a 95% pointwise confidence interval based on Aalen's additive model.

The covariates in model 4 (Table S1) were used to fit the Aalen additive hazard model. The result is comparable to the Cox PH model in identifying the risk factors of graft survival (Table 4). However, the Kolmogorov-Smirnov test shows some evidence of time-varying effect for Donor type and recipient ethnicity in this table (*p*-values < 0.05), this is supported by Von Cramer Mises test (result not included). The plot of the cumulative regression coefficients for the Aalen model is shown in Figure 4. There is a linear increase in the hazard of graft failure with an increase in the recipient age and its confidence interval does not include the zero line, indicating that age has a significant effect on the hazard of graft failure over the years of follow-up. The 95% confidence interval for other plots include the zero line at some time point, indicating covariates with early (e.g., Delayed graft function) and late (donor type) significant effects on graft survival. Only the plot for donor type has a negative effect on graft survival, the effect at some points flattens before it steeply decreases linearly, which by the test is an indication of a time-varying effect. The cumulative plot for recipient ethnicity shows a curvilinear pattern, it displays a steep increase at the beginning of the follow-up and shows a roughly zero slope after the first 10 years. The plot suggests a time-varying effect for recipient ethnicity and also that this covariate may not have a late significant effect on graft survival.

**Table 4:** Tests for non-significant and time-varying effects of the covariates in the Aalen additive hazard model.

**Covariates**	**Non-significant effects**		**Time invariant effects**	
	**Statistics**	***p*-value**	**Statistics**	***p*-value**
Intercept	3.01	0.061	0.19	0.730
Donor type	3.86	0.003	0.27	0.035
Delayed graft function	5.06	<0.001	0.23	0.217
Diabetes at transplant	3.37	0.018	0.34	0.389
Recipient ethnicity	5.01	<0.001	0.26	0.043
Recipient age	5.91	<0.001	0.01	0.518
Donor-recipient gender (f-f)	3.26	0.023	0.22	0.434
Donor-recipient gender (f-m)	2.68	0.163	0.21	0.370
Donor-recipient gender (m-f)	1.87	0.688	0.27	0.303

**Figure 5:**
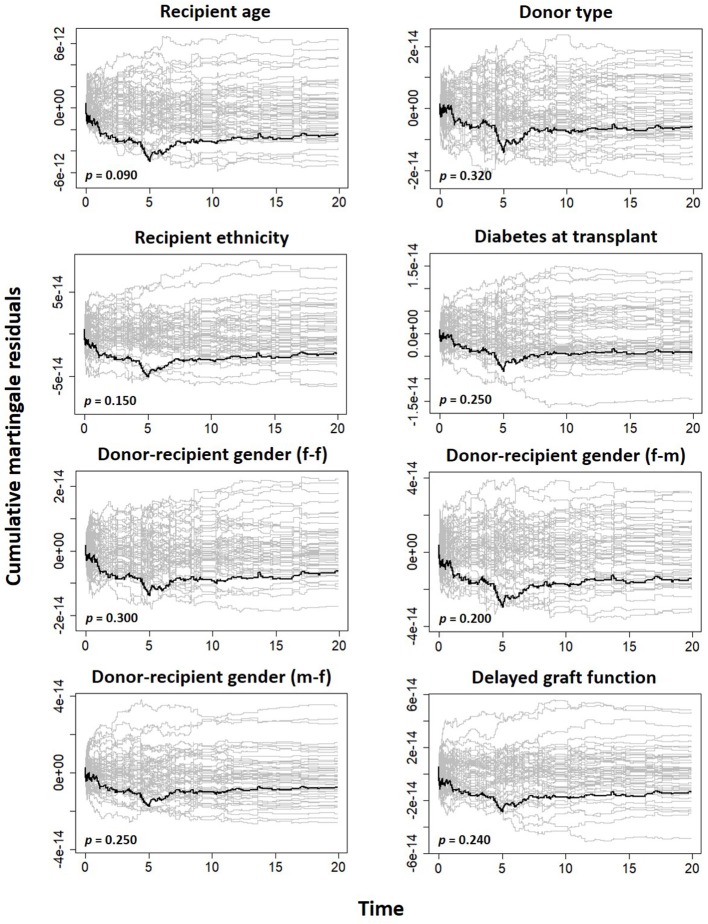
Plot of cumulative martingale residual from Aalen additive model.

The cumulative martingale residual together with 50 simulated processes (Figure 5) under the Aalen model shows that the covariates' behavior is more typical with the model (*p*-values > 0.05), indicating a good fit of Aalen model. The result of the semi-parametric version of the Aalen model is shown in Table 5, all the covariates as previously reported shows significant effects on graft survival. For the covariates with constant effects as suggested by the Aalen model, their estimates are shown in Table 5.

**Table 5:** Analysis of risk factors based on the Cox PH, McKeague and Sasieni hazard, and Weibull AFT models.

**Variable**	**Cox PH model**		**Additive hazard model**		**Weibull model**	
	**HR (95% CI)**	***p*-value**	**Coefficient (se)**	***p*-value**	**TR (95% CI)**	***p*-value**
Recipient age	1.03 (1.02–1.04)	<0.001	0.0023 (0.0004)	<0.001	0.95 (0.93–0.97)	<0.001
Donor type						
Cadaveric	1					
Living	0.62 (0.43–0.90)	0.012		0.001	2.40 (1.26–4.57)	0.008
Recipient ethnicity						
White	1					
Non-white	1.50 (1.22–1.85)	<0.001		<0.001	0.49 (0.34–0.70)	<0.001
Diabetes at transplant						
No	1		…			
Yes	1.59 (1.12–2.28)	0.010	0.0299 (0.0165)	0.054	0.45 (0.24–0.83)	0.011
Donor-recipient gender						
m-m	1		…			
f-f	1.48 (1.09–2.02)	0.013	0.0327 (0.0136)	0.026	0.44 (0.26–0.76)	0.003
f-m	1.25 (0.97–1.60)	0.082	0.0169 (0.0101)	0.095	0.66 (0.43–1.02)	0.060
m-f	1.16 (0.87–1.55)	0.321	0.0123 (0.0116)	0.284	0.72 (0.43–1.19)	0.196
Delayed graft function						
No	1		…			
Yes	1.49 (1.21–1.85)	<0.001	0.0355 (0.0104)	0.001	0.49 (0.34–0.71)	<0.001

*Based on the Aalen's additive hazard model, Donor type, and Recipient ethnicity have time-varying effects on graft survival, and their effects are not estimated under McKeague and Sasieni hazard model*.

### 4.4. Result From Parametric AFT Models

**Figure 6:**
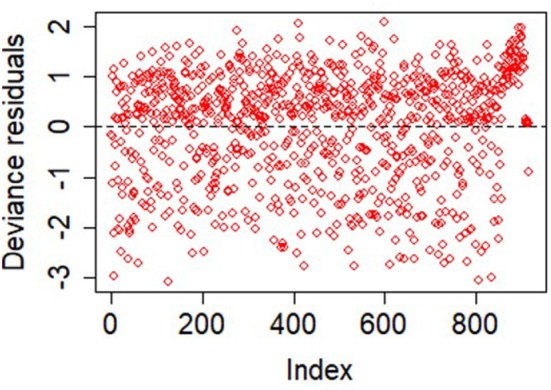
Assessment of goodness-of-fit using the plots of the deviance residuals.

The AIC values of the models are 2,444, 2,492, and 2,471 for Weibull, lognormal, and log-logistic models, respectively. The rule is that any model that conforms to the observed data should adequately lead to a smaller AIC. Based on this, the Weibull model is the best-performing model. The distribution of the deviance residuals from the Weibull model is mostly within the range of ±3 except three observations that are slightly outside this bound (Figure 6). The result of the Weibull model is similar to that of the Additive hazard models in detecting the significant predictors of graft survival and their directional effects (positive or negative effect), although, the interpretations are not the same. For instance, in Table 5, the semi-parametric additive hazard model shows that female patients that received a kidney from female donors had an increase in the hazard of 0.0327 compared with male patients that received a kidney from male donors. Conversely, the Weibull model shows that female recipients of a kidney from female donors had 44% lower graft survival in comparison to male patients that received from male donors. The Weibull models show that the influence of all the predictors except donor type, decelerate graft survival time. Every additional increase in age, on the average age of the recipients, is associated with 5% decrease in graft survival, this indicates that the older the patient, the higher the hazard of graft failure. This is similar to what is observed in the additive hazard model. Also, the results show that graft survival is prolonged (more than twice) among patients that received live kidney transplant compared with those that received a cadaveric kidney transplant.

## 5. Discussion

In this study, 915 adult patients that underwent a kidney transplant at Charlotte Maxeke Academic Hospital Johannesburg, South Africa were analyzed. This study attempts to appropriately employ more statistically justifiable strategies in selecting the best combination of predictors that influence long-term GS post-kidney transplantation. The method of imputation used in this study has been shown (in studies using different biological and medical datasets) to outperform imputation methods, such as multivariate imputation by the chained equation, nearest neighbor and mean imputation (20, 31). The Cox PH model is the most attractive survival model when a set of covariates are of interest in modeling time to graft failure. Fitting a large number of variables in this model could add noise to the estimated quantities, resulting in collinearity among variables and increase the cost of modeling unnecessary predictors. The purposeful variable selection method based on the Cox PH model becomes more complex when there are too many predictors in the data. However, this procedure of model building involves a combination of science, statistical method, experience and common sense (32). The purposeful method has been applied in previous studies (5, 23, 32). These studies comparatively showed that purposeful variable selection method leads to significant variables, confounding factors and a richer model compared with other selection methods; when prediction and identification of risk factors are of interest.

Evaluating the PH assumption for all predictors in the Cox PH model should be a fundamental aspect of the modeling process when using the Cox PH model. Including variable(s) not satisfying the PH assumption leads to an inferior fit of a Cox model i.e., the power of tests is reduced for both variables with constant and non-constant HR in the model. Our results provide evidence of time-varying effects for the covariates in the Cox PH model. This shows that it is necessary to assess this assumption based on the fact that clinical variables effects are rarely constant over time.

The Cox PH, additive hazard and AFT models are used in survival to study the association between risk factors and the event of interest in failure time data. The appropriateness of the individual model may not be known in advance for a specific application. The models may capture the risk process equally or sometimes give a different result (10, 29, 33). For many application in public health, the additive hazard may be plausible since the result gives differences in hazard, rather than a hazard ratio. The same applies to the straightforward interpretation of TR as compared to HR. These models may be compared with regards to the *p*-values of the covariates in the model, since the greatness of *p*-value shows the power to reject the null hypothesis (10).

We identified that factors, such as “donor age” and “acute rejections” previously shown to be important risk factors of GS (34–36) are neither significant nor confounders in this study. The difference between the findings of this present study and these previous studies could be linked to differences in sample size (number of graft failures observed), year of transplant, duration of follow-up and method of data analysis. Nevertheless, it is noteworthy that the significant predictors of GS observed in this study are in agreement with previous studies (11, 16–18, 34, 35, 37, 38).

Prognostic assessment with the Cox PH model is generally based on patients/donors characteristics at the time of evaluation. These characteristics have a greater tendency to change, following a long period of study. We have shown in this study that when long-term follow-up is of interest, survival prediction may be discordant with the Cox PH model. We have statistically shown that the Cox PH model did not capture all the significant aspects of the data analysis and did not provide adequate fit in this study. We were able to investigate the time-varying covariate effects with the Aalen additive model and fully estimates the effects of the covariate with the AFT model. The need to explore beyond the Cox PH model is revealed in the Aalen plot, the plot can aide a nephrologist to understand the pattern by which the covariates influence graft survival after transplantation. Considering censored quantile regression model could be alternatives when the PH assumption is not valid in the Cox PH model.

This study has several important strengths. We have used a rational approach in analyzing the kidney transplant data generated from a South African transplant cohort study. The results of this historical data analysis could help to understand long-term performance and progress of kidney transplant outcome in this region, and how the risk factors influence the survival of the graft after kidney transplant. The analysis involves a combination of both recipients of a cadaveric and living donor kidney transplant, focusing on graft failure because maximizing graft is paramount important to the recipient of a new kidney and transplant unit. We found in this study that predictors of graft survival may exhibit time-varying effects.

On the other hand, this study also has some methodological limitations. We found that multicollinearity is a problem in using the purposeful method of variable selection, especially when the covariates are highly related. Specifically, we noticed that dropping any of the causes of ESKD influences the coefficients of others. Taking a decision on which variable to add or retain some times is challenging. However, because the procedure is governed by a specific rule at each step, the choice or decision to drop or retain any variable was critically assessed to avoid multicollinearity in the final model. In addition, 57% censoring observed is another limitation in this study.

## 6. Conclusion

Additive hazard and AFT models are yet to receive more deserving attention in modeling GS after a kidney transplant. When covariate effects involve certain patterns of heterogeneity in kidney transplant studies, additive hazard and AFT models could offer great flexibility in modeling GS time. The models used in this study describe different features of the relationship between the risk factors and graft failure. Hence, it appears necessary to use these models complementarily to gain a more comprehensive understanding of GS after a kidney transplant.

## Data Availability

The datasets generated for this study are available on request to the corresponding author.

## Ethics Statement

Ethical clearance for JF, approved by the Wits Human Research Ethics Committee. Medical clearance certificate number: M121186.

## Author Contributions

OA performed the data cleaning and analysis, interpreted the ensuing results, wrote, and edited the manuscript. JF provided the data used for this study, supervised the data cleaning steps, and edited the manuscript. EM supervised the analysis and the manuscript production, and edited the manuscript.

### Conflict of Interest Statement

The authors declare that the research was conducted in the absence of any commercial or financial relationships that could be construed as a potential conflict of interest.
