# Supplementary material for: Modeling Long-Term Graft Survival With Time-Varying Covariate Effects: An Application to a Single Kidney Transplant Centre in Johannesburg, South Africa
Source: Front Public Health. 2019 Jul 25;7:201. doi: 10.3389/fpubh.2019.00201 (PMC6669915; doi:10.3389/fpubh.2019.00201)
Supplement: Supplementary file 1 [file Data_Sheet_1.PDF]

Supplementary Material - long-term graft survival with time-varying covariate effects: An application to a single kidney transplant centre in Johannesburg, South Africa

July 3, 2019

1 Supplementary Tables and Figures

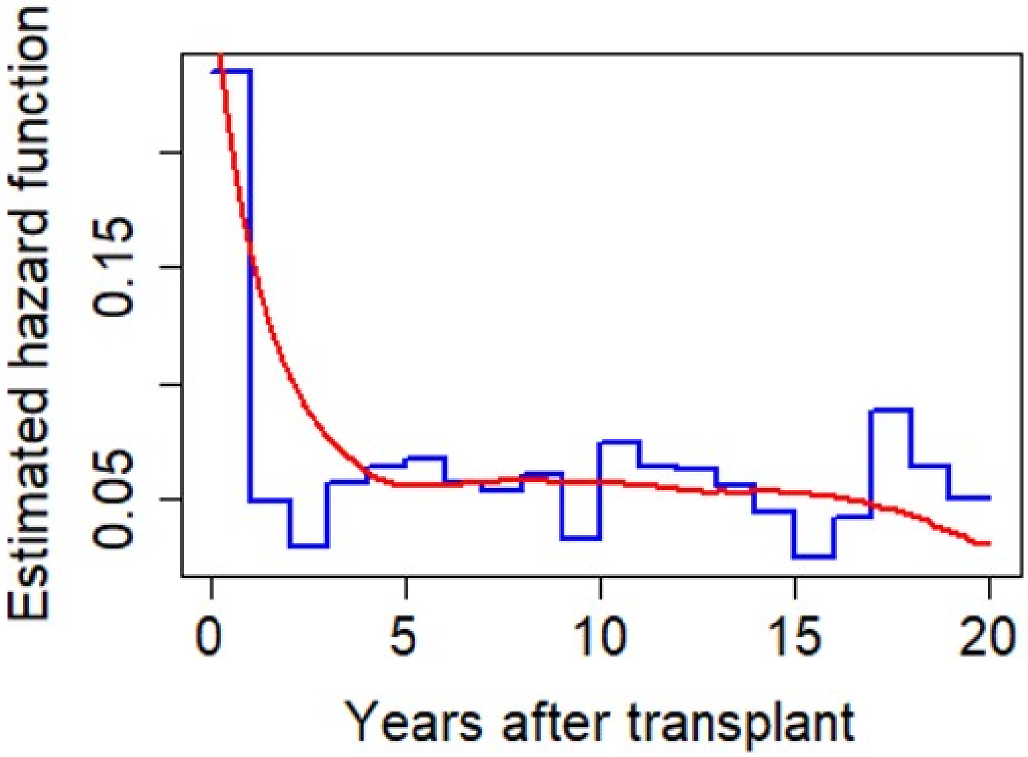

Figure S1: Kaplan-Meier estimates of a kernel-smoothed hazard function

Table S1: Summary of the four models described in Table 2

Model	Variables	HR(95% CI)	*p*-value
	Donor type	0.68(0.46-0.99)	0.045
	Renal ESKD	0.80(0.59-1.08)	0.140
	Hypertension ESKD	0.83(0.61-1.12)	0.220
	Urological ESKD	0.76(0.48-1.21)	0.249
	Inherited ESKD	0.65(0.42-0.99)	0.047
**1**	Surgical complication	0.86(0.69-1.06)	0.152
	Delayed graft function	1.39(1.11-1.73)	0.003
	Diabetes at transplant	1.57(1.09-2.26)	0.017
	Recipient ethnicity	1.44(1.12-1.86)	0.004
	Donor age	1.00(0.99-1.01)	0.654
	Recipient age	1.03(1.02-1.04)	<0.001
	Histological acute rejection	1.17(0.85-1.60)	0.330
	Donor type	0.66(0.46-0.95)	0.026
	Inherited ESKD	0.78(0.54-1.13)	0.182
	Surgical complication	0.86(0.69-1.06)	0.160
**2**	Delayed graft function	1.41(1.14-1.75)	0.002
	Diabetes at transplant	1.53(1.07-2.18)	0.018
	Recipient ethnicity	1.45(1.17-1.78)	0.001
	Recipient age	1.03(1.02-1.04)	<0.001
	Donor type	0.66(0.45-0.95)	0.025
	Delayed graft function	1.46(1.18-1.81)	<0.001
**3**	Diabetes at transplant	1.57(1.10-2.23)	0.012
	Recipient ethnicity	1.49(1.21-1.83)	<0.001
	Recipient age	1.03(1.02-1.04)	<0.001
	Donor type	0.62(0.43-0.90)	0.012
	Delayed graft function	1.49(1.21-1.85)	<0.001
	Diabetes at transplant	1.59(1.12-2.28)	0.010
**4**	Recipient ethnicity	1.51(1.22-1.85)	0.000
	Recipient age	1.03(1.02-1.04)	<0.001
	Donor-recipient gender *(f-f)*	1.48(1.09-2.02)	0.013
	(f-m)	1.25(0.97-1.60)	0.082
	(m-f)	1.16(0.87-1.55)	0.320

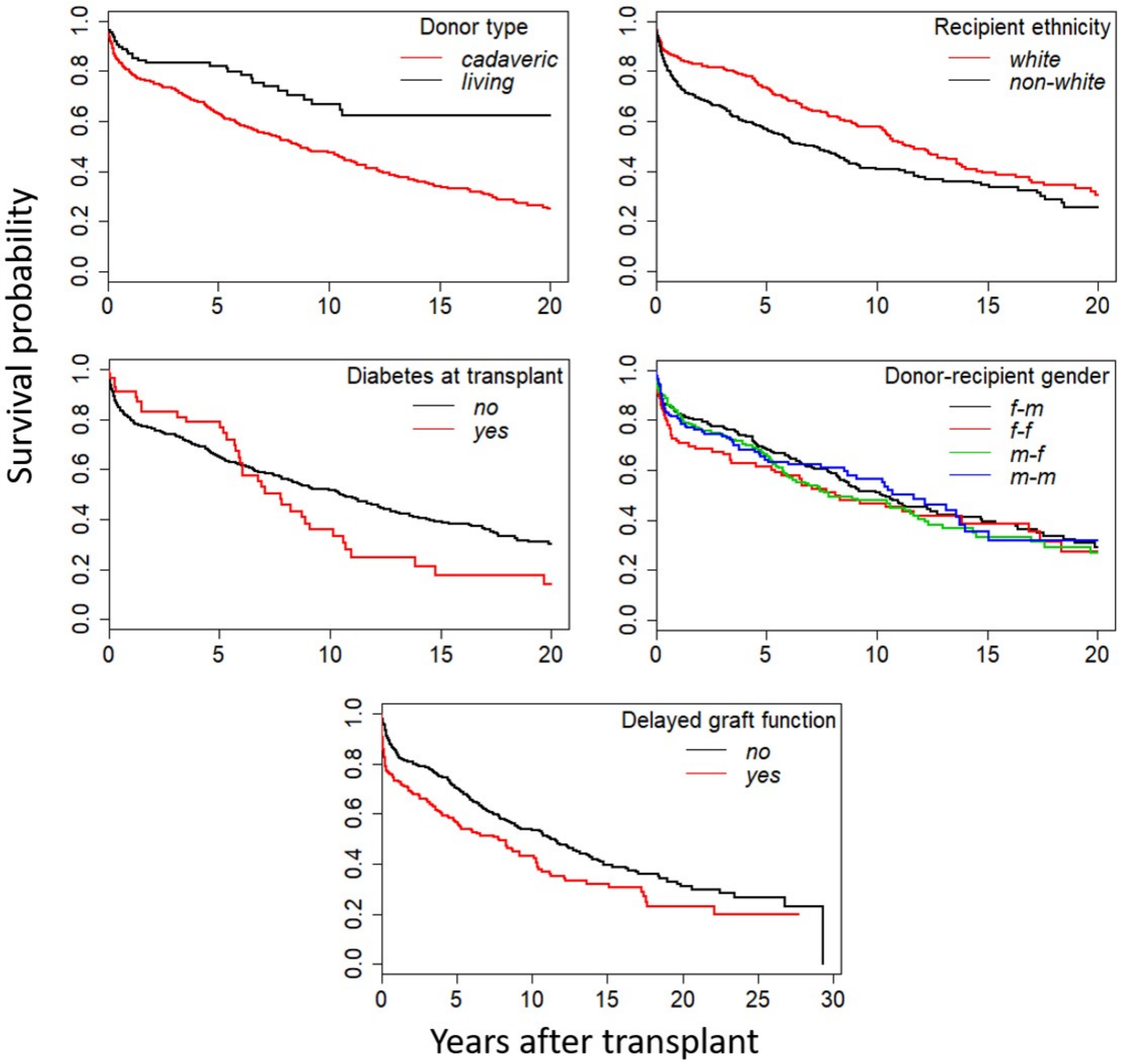

Figure S2: Plot of graft survival probability vs. years post transplant for the categorical variables in Model 4

Table S2: Result of the extended Cox PH model with main effects of the covariates with time-varying effects and their interaction with time

**Variable**	**HR**	***p*-value**
Donor type	0.63	0.017
Delayed graft function	1.73	<0.001
Diabetes at transplant	1.18	0.537
Donor-recipient gender		
f-f	1.47	0.016
f-m	1.22	0.120
m-f	1.14	0.371
Recipient ethnicity	1.84	<0.001
Recipient age	1.03	<0.001
(Delayed graft function) $\times$ time	1.04	0.131
(Diabetes at transplant) $\times$ time	0.95	0.113
(Recipient ethnicity) $\times$ time	1.05	0.036
**Proportionality test**		**0.083**

Table S3: Comparing variables selected with the purposeful method of variable selection and the automated methods

Variables	Purposeful	Stepwise	Backwards	Forward	Best subset
Recipient age	✓	✓	✓	✓	✓
Donor type	✓	✓	✓	✓	✓
Recipient ethnicity	✓	✓	✓	✓	✓
Diabetes at transplant	✓	✓	✓	✓	✓
Donor-recipient gender	✓	✓	✓	✓	✓
Delayed graft function	✓	✓	✓	✓	✓
Inherited ESKD	✗	✓	✓	✓	✓
Surgical complication	✗	✗	✗	✗	✓

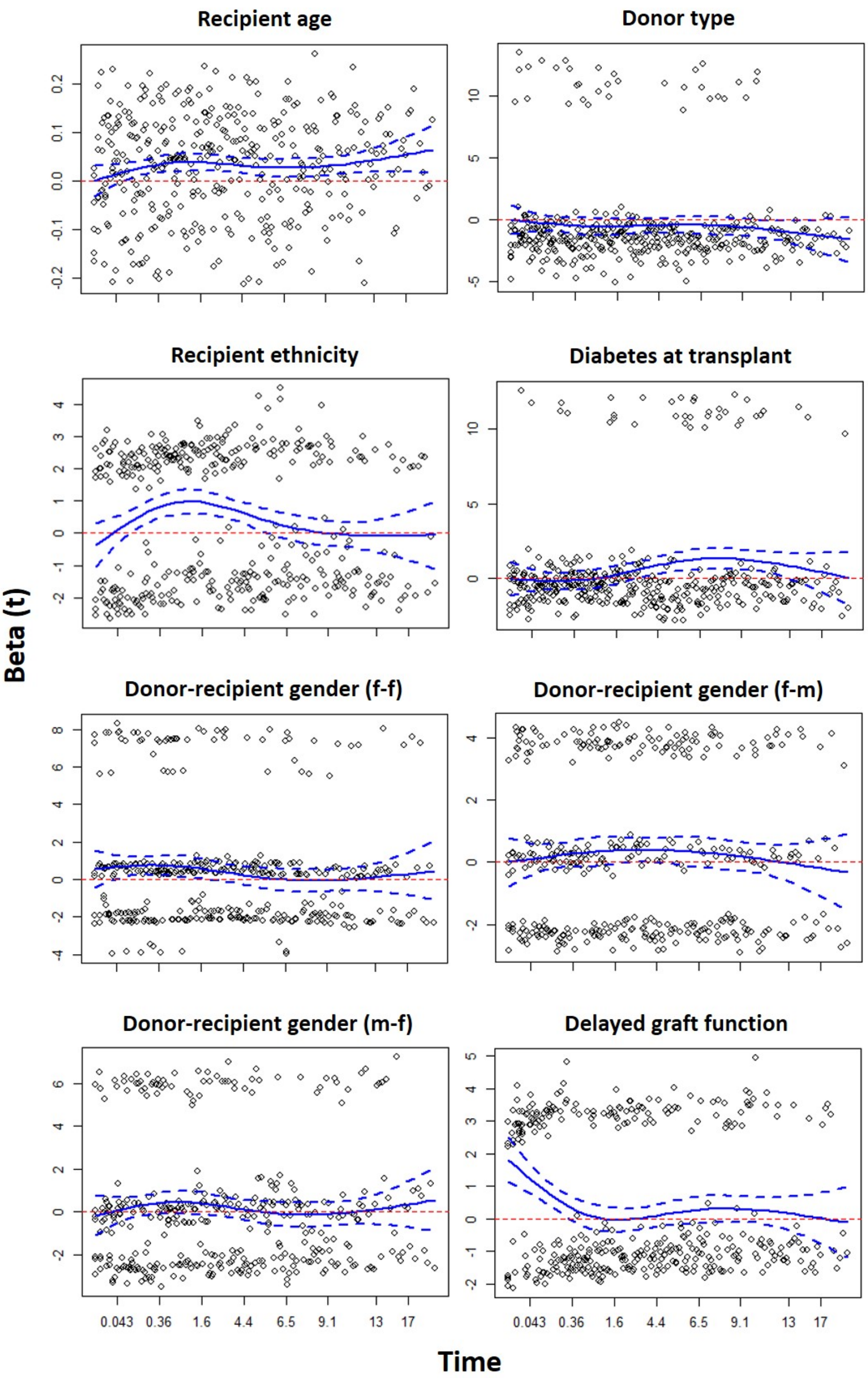

Figure S3: Assessing of PH assumption: graphs of the scaled Schoenfeld residuals versus transformed time for each covariate in the Cox PH model. The solid and the broken lines represent the smoothing spline fit and the ±2 standard error for the fit.

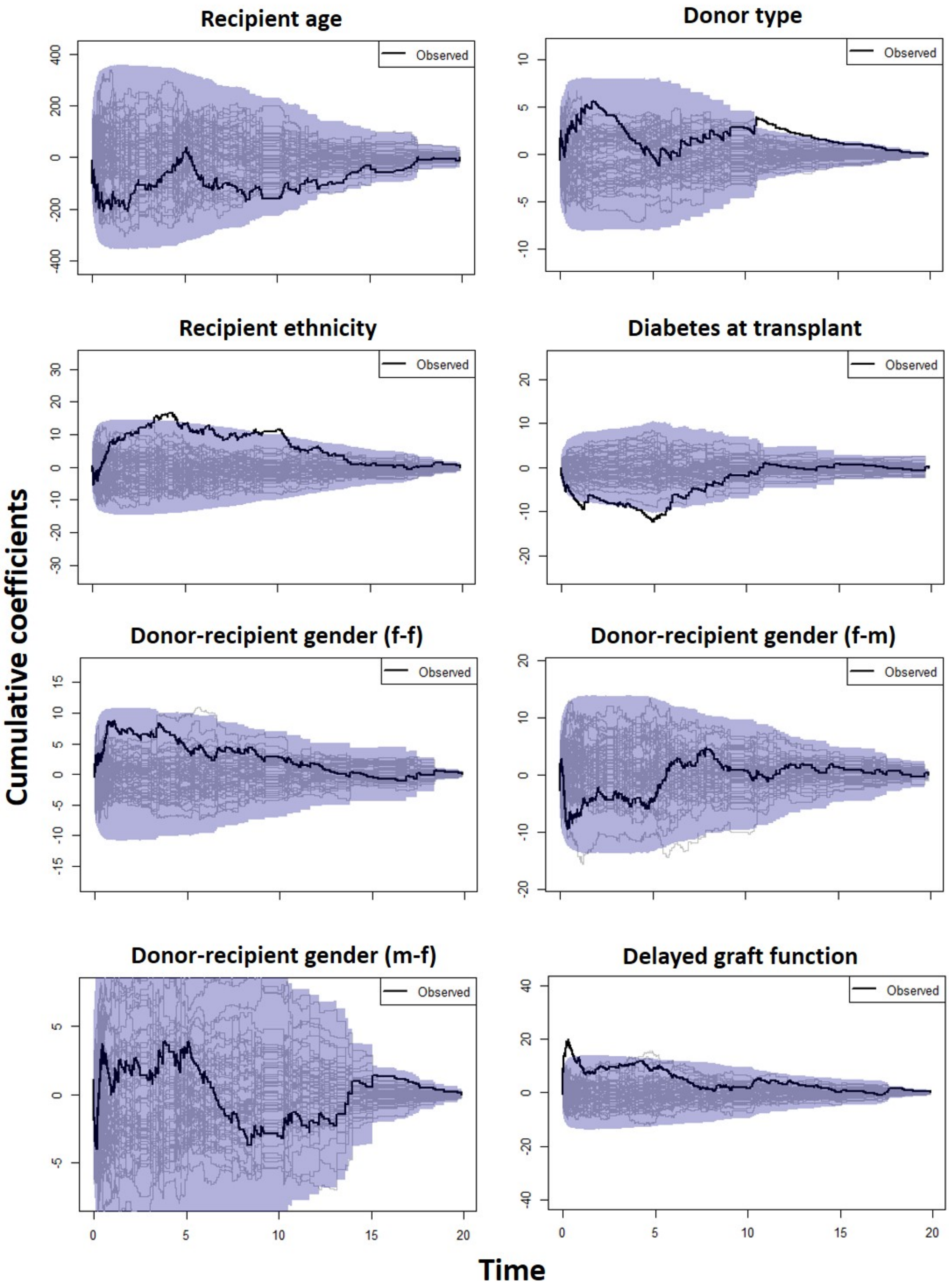

Figure S4: Assessing of PH assumption:graphs of observed test processes with 50 simulated processes for each covariate in the Cox PH model. The solid black profile signifies the observed pattern.

2 R codes

```
#R codes for analyses
#===reading in the libraries
rm(list=ls())
library(survival)
library(gof)
library(BaylorEdPsych)
library(dplyr)
library(missForest)
library(survminer)
library(muhaz)
library(MASS)
library(bshazard)
library(ISLR)
library(leaps)
library(timereg)
library(flexsurv)
#===importing data from a csv file
CYA <- read.csv("C:/Users/Ike/Desktop/MSc_data/CYA.csv", header =TRUE)
str(CYA)
summary(CYA)

#===Data manipulation after several steps of data cleaning
CYA2 <- CYA
CYA2$ethnicity<-factor(CYA2$ethnicity)
CYA2$new_bloodgroup<-factor(CYA2$new_bloodgroup)
CYA2$new_gender<-factor(CYA2$new_gender)
CYA2$ethnicity <- relevel(CYA2$ethnicity, ref="white")
CYA2$new_gender <- relevel(CYA2$new_gender, ref="male-male")
CYA2$diabetes<-CYA2$diabetes_tx
CYA2$recip_age<-CYA2$Recip_Age_cont
CYA2 <- CYA2[c(-3,-9,-10,-11,-12,-15)]
names(CYA2)

#===arrange variables
CYA2<-CYA2 %>%dplyr:: select(job_id,Grftsurvtime, Graftstatus,recip_age,
renal_disease,hypertension, urological,inherited, AR_clinical,
AR_histological,everything())
```

```
#===Missing completely at random test
Mcar<-LittleMCAR(data.frame(CYA2[4:18]))

Mcar[c("chi.square", "df", "p.value")]

#===subset data to 20 years because of few survival recorded
 towards the end of the study
CYA_20<-CYA2
CYA_20$Grftsurvtime[CYA2$Grftsurvtime>20]<-20
CYA_20$Graftstatus[CYA2$Grftsurvtime>20]<-0
surv20<-with(CYA_20, Surv(Grftsurvtime,Graftstatus))
summary(CYA2$Grftsurvtime)
surv_rate<-survfit(Surv(Grftsurvtime, Graftstatus)~ 1, data=CYA_20)
summary(surv_rate, times=c(1,5,10,15,20))



#===Extract dates of transplant & survival variables, Fig2A
timeD<-read.csv("C:/Users/Ike/Desktop/timeseries.csv", header = TRUE)
time_series<-subset(timeD,timeD$Transplant_era=="CYA")
time_series2<-as.Date(time_series$date_tx,"%m/%d/%Y")
class(time_series2)
time_series3<-format(time_series2, format="%Y")
time_series_F<-cbind(time_series,time_series3)

time_series_T<-table(time_series_F$time_series3)
time_series_D<-data.frame(time_series_T)
head(time_series_D)
barplot(Freq~Var1, data = time_series_D,cex.lab=1.5,cex.axis=1.5,
xlab = "Years of transplant",ylab = "Frequency",cex.names=1.5)

#===Histogram and survival curve, Fig. 2B and C
trans_km <-survfit(Surv(CYA_20$Grftsurvtime, CYA_20$Graftstatus)~1)
trans_km
plot(trans_km)
summary(trans_km,times=1)
par(mfrow=c(2,2))
his_pre<-hist(CYA_20$Grftsurvtime,xlab = "Years after transplant",
main = NULL, col="gray", ylim=c(0,400),cex.lab=1.5, cex.axis =1.5)
plot(trans_km,xlab="Years after transplant", ylab= "Survival
probability",  mark.time = T, col="black", conf.int =F,cex.lab=1.5, cex.axis =1.5)
```

```
#===Hazard rate estimates,Fig 2D
uni<- bshazard(Surv(Grftsurvtime,Graftstatus)~1, CYA_20)
plot(uni,col="blue", lwd=2, ylab = "Estimated hazard function",
xlab="Years after transplant",
cex.lab=1.5, cex.axis =1.5)
#plot(uni)
plot(uni$time,uni$hazard*1000,col="black", type = "l", lwd = 3,
ylab = "Graft failure rate per 1000 PY", xlab="Years after transplant",
cex.lab=1.5, cex.axis =1.5)

#===Imputation
set.seed(81)
for_imp <- missForest(mspap, verbose = TRUE,maxiter = 5, ntree = 500)
for_imp$OOBerror
imputed<-for_imp$ximp
is.na(imputed)
imputed<-imputed %>%dplyr:: select(job_id,Grftsurvtime,Graftstatus,
recip_age, dnr_age, everything())
names(imputed)

#===univariate analysis
uni_all<-colnames(imputed)[4:18]
for (i in 1:length(uni_all)){
print(uni_all[i])
print(coxph(Surv(Grftsurvtime,Graftstatus) ~ get(uni_all[i]), data=imputed))
}
for_sur<-with(imputed, Surv(Grftsurvtime,Graftstatus))

#===centering
imputed$recip_age3<- scale(imputed$recip_age, center = TRUE,scale = FALSE)
imputed$dnr_age3<- scale(imputed$dnr_age, center = TRUE,scale = FALSE)

#===purposeful method of variable selection===#
purv2<-coxph(for_sur~donor_type+renal_disease+hypertension+
urological+inherited+no_complication+
delayed_gf+diabetes+ethnicity+dnr_age3+recip_age3+AR_histological,
data=imputed)

summary(purv2)
-2*purv2$loglik[2]
```

```
#===drop dnrage
purv3<-coxph(for_sur~donor_type+renal_disease+hypertension+urological
+inherited+no_complication+
delayed_gf+diabetes+ethnicity+recip_age3+AR_histological,data=imputed)
summary(purv3)
-2*purv3$loglik[2]
# likelihood ratio test:
X.lr3=-2*purv3$loglik[2]-(-2*purv2$loglik[2]) # test statistics
X.lr3
1-pchisq(X.lr3,1) # p-value
delta.coff<-abs((coef(purv3)-coef(purv2)[-10])/coef(purv2)[-10])
round(delta.coff,5)

#===drop AR_histological
purv4<-coxph(for_sur~donor_type+renal_disease+hypertension+urological
+inherited+no_complication+
delayed_gf+diabetes+ethnicity+recip_age3,data=imputed)
summary(purv4)
-2*purv4$loglik[2]
# likelihood ratio test:
X.lr4=-2*purv4$loglik[2]-(-2*purv3$loglik[2]) # test statistics
X.lr4
1-pchisq(X.lr4,1) # p-value
delta.coff<-abs((coef(purv4)-coef(purv3)[-11])/coef(purv3)[-11])
round(delta.coff,5)

#===urological,renal_d and hyper were dropped because of urological influence
purv5<-coxph(for_sur~donor_type+renal_disease+hypertension+inherited
+no_complication+
delayed_gf+diabetes+ethnicity+recip_age3,data=imputed)
summary(purv5)
-2*purv5$loglik[2]
X.lr5=-2*purv5$loglik[2]-(-2*purv4$loglik[2])
X.lr5
1-pchisq(X.lr5,1) # p-value
delta.coff<-abs((coef(purv5)-coef(purv4)[-4])/coef(purv4)[-4])
round(delta.coff,5)


#===restart the model building process
purv6<-coxph(for_sur~donor_type+inherited+no_complication+
```

```
delayed_gf+diabetes+ethnicity+recip_age3,data=imputed)
summary(purv6)
-2*purv6$loglik[2]

#===inherited
purv7<-coxph(for_sur~donor_type+no_complication+
delayed_gf+diabetes+ethnicity+recip_age3,data=imputed)
summary(purv7)
-2*purv7$loglik[2]
# likelihood ratio test:
X.lr7=-2*purv7$loglik[2]-(-2*purv6$loglik[2]) # test statistics
X.lr7
1-pchisq(X.lr7,1) # p-value
delta.coff7<-abs((coef(purv7)-coef(purv6)[-2])/coef(purv6)[-2])
round(delta.coff7,5)

#===nocomplication
purv7a<-coxph(for_sur~donor_type+
delayed_gf+diabetes+ethnicity+recip_age3,data=imputed)
summary(purv7a)
-2*purv7a$loglik[2]
# likelihood ratio test:
X.lr7a=-2*purv7a$loglik[2]-(-2*purv7$loglik[2]) # test statistics
X.lr7a
1-pchisq(X.lr7a,1) # p-value
delta.coff7<-abs((coef(purv7a)-coef(purv7)[-2])/coef(purv7)[-2])
round(delta.coff7,5)


#===Add variables not sig at 25%===#
#===gender
purv8<-coxph(for_sur~donor_type+
delayed_gf+diabetes+ethnicity+recip_age3+new_gender,data=imputed)
summary(purv8)

-2*purv8$loglik[2]
# likelihood ratio test:
X.lr8=-2*purv7a$loglik[2]-(-2*purv8$loglik[2]) # test statistics
X.lr8
1-pchisq(X.lr8,1) # p-value
#delta.coff8<-abs((coef(purv7)-coef(purv8)[-7])/coef(purv8)[-7])
```

```
#round(delta.coff8,5)

#===AR_histological, bloodgroup
purv9<-coxph(for_sur~donor_type+
delayed_gf+diabetes+ethnicity+recip_age3+new_gender+new_bloodgroup,
data=imputed)
summary(purv9)
-2*purv9$loglik[2]
X.lr9=-2*purv8$loglik[2]-(-2*purv9$loglik[2]) # test statistics
X.lr9
1-pchisq(X.lr9,1) # p-value
delta.coff9<-abs((coef(purv8)-coef(purv9)[-7])/coef(purv9)[-7])
round(delta.coff9,5)

#===Final model====#
Finover<-coxph(for_sur~donor_type+
delayed_gf+recip_age3+diabetes+new_gender+ethnicity,data=imputed)
summary(Finover)
vif(Finover)

#===Linearity assessment
smooth_SEcurve<-function(yy,xx){
list_x<-min(xx) + ((0:100)/100)*(max(xx)-min(xx))
yy_xx<-predict(loess(yy~xx),se=T,newdata=data.frame(xx=list_x))
lines(yy_xx$fit ~list_x, lwd=2,col="blue")
lines(yy_xx$fit - qt(0.975,yy_xx$df)*yy_xx$se.fit~list_x, lty=2)
lines(yy_xx$fit + qt(0.975,yy_xx$df)*yy_xx$se.fit~list_x, lty=2)
}
mart <-coxph(Surv(imputed$Grftsurvtime, imputed$Graftstatus)~1)
martR <-residuals(mart, type="martingale")
par(mfrow=c(2,2))
plot(martR~imputed$recip_age,xlab="recip_age", ylab = "Martingale
residual", col="red", cex.lab=1.5, cex.axis =1.5)
smooth_SEcurve(martR,imputed$recip_age)
#pspline
ps_cox <-coxph(for_sur~donor_type+delayed_gf+diabetes+ethnicity+pspline
(recip_age3,df=4)
+new_gender,data=imputed)
ps_cox
termplot(ps_cox,se=T, terms = 1,ylabs = "Log hazard", col.term = "blue",
col.se = "blue",cex.lab=1.5,
```

```
cex.axis =1.5,lwd.term = 2,lwd.se = 2)

#===PH assessment
cox.zph(Finover)

par(mfrow=c(2,2))
plot(cox.zph(Finover),ann=T, var=1,col="blue",lwd=2)
abline(h=0, lty=2, col="red")
plot( cox.zph(Finover), ann=T, var=2, col="blue",lwd=2 )
abline(h=0, lty=2, col="red")
plot( cox.zph(Finover), ann=T, var=3 , col="blue",lwd=2)
abline(h=0, lty=2, col="red")
plot( cox.zph(Finover), ann=T, var=4, col="blue",lwd=2)
abline(h=0, lty=2, col="red")
plot( cox.zph(Finover), ann=T, var=5, col="blue",lwd=2)
abline(h=0, lty=2, col="red")
plot( cox.zph(Finover), ann=T, var=6,col="blue", lwd=2)
abline(h=0, lty=2, col="red")
plot( cox.zph(Finover), ann=T, var=7,col="blue", lwd=2)
abline(h=0, lty=2, col="red")
plot( cox.zph(Finover), ann=T, var=8,col="blue", lwd=2)

cmr <- cumres(Finover,R=50)
cmr
par(mfrow=c(2,2))
plot(cmr,legend = c("type2"))
abline(h=0, lty=2, col="red")

par(mfrow=c(2,2))
KM1= survfit(Surv(Grftsurvtime, Graftstatus)~ donor_type, data=imputed)
plot(KM1,lwd =2, xlab="Years after transplant",ylab= "Survival
probability", col=c("red","black"), conf.int = F,cex.lab=1.5, cex.axis =1.5)
legend("topright",c("cadaveric","living"),col=c("red","black"),lty=1,bty='n',
title = "Donor type",text.font = 3,cex = 1.5)

KM2 = survfit(Surv(Grftsurvtime, Graftstatus)~ ethnicity, data=imputed)
plot(KM2,lwd =2,  xlab="Years after transplant",ylab= "Survival probability",
col=c("red","black"), conf.int = F,cex.lab=1.5, cex.axis =1.5)
legend("topright",c("white", "non-white"),col=c("red","black"),lty=1,bty='n',
title = "Recipient ethnicity",text.font = 3,cex = 1.5)
```

```
KM3 = survfit(Surv(Grftsurvtime, Graftstatus)~ delayed_gf, data=imputed)
plot(KM3,lwd =2,  xlab="Years after transplant",ylab= "Survival probability",
col=1:2, conf.int = F,cex.lab=1.5, cex.axis =1.5)
legend("topright",c("no","yes"),col=1:2,lty=1,bty='n',title = "Delayed graft
function",text.font = 3,cex = 1.5)

KM4= survfit(Surv(Grftsurvtime, Graftstatus)~ diabetes, data=imputed)
plot(KM4,lwd =2,  xlab="Years after transplant",ylab= "Survival probability",
col=1:2, conf.int = F,cex.lab=1.5, cex.axis =1.5)
legend("topright",c("no","yes"),col=1:2,lty=1,bty='n',title = "Diabetes at
transplant",text.font = 3,cex = 1.5)

KM4= survfit(Surv(Grftsurvtime, Graftstatus)~ new_gender, data=imputed)
plot(KM4,lwd =2,  xlab="Years after transplant",ylab= "Survival probability",
col=1:4, conf.int = F,cex.lab=1.5, cex.axis =1.5)
legend("topright",c("f-m","f-f","m-f","m-m"),col=1:4,lty=1,bty='n',title =
"Donor-recipient gender",
text.font = 3,cex = 1.5)

#===overall Cox model fit
coxsnellres=imputed$Graftstatus-resid(Finover, type="martingale")
fitres=survfit(coxph(Surv(coxsnellres, imputed$Graftstatus)~1, method
= 'breslow'),type='aalen')
fitres
plot(fitres$time, -log(fitres$surv), type = 'p', xlab = ' Cox-snell residuals',
ylab ='Estimated cumulative hazard function',lwd=2.5,lty=6,cex.lab=1.5,
cex.axis =1.5)
abline(0,1, col='red', lwd=2,lty=1)
par(mfrow=c(2,2))

dev_cox<-residuals(Finover,type="deviance")
plot(dev_cox,col="red",ylab="Deviance residual for Cox PH model",cex.lab=1.5,
cex.axis =1.5)
abline(h=0, lty=2)
identify(dev_cox)

#===Additive hazard===
Finover4<-aalen(for_sur~donor_type+delayed_gf+diabetes+ethnicity+recip_age
+new_gender,max.time = 20, residuals = 1,data=imputed)
Finover5<-aalen(for_sur~donor_type+const(delayed_gf)+const(diabetes)+ethnicity+
const(recip_age)+const(new_gender),residuals=1,data=imputed)
```

```
Finover4
Finover5
par(mfrow=c(2,2))
plot(Finover4,cex.lab=1.5, cex.axis =1.5,lwd=2)

#==cumulative martingal residual
par(mfrow=c(2,2))
X<-model.matrix(~donor_type+delayed_gf+diabetes+ethnicity+recip_age3+
new_gender,data=imputed,residuals=1,n.sim=100)
resids<-cum.residuals(Finover4,cum.resid=1,modelmatrix=X,n.sim=100,data=imputed)
plot(resids,score=1,cex.lab=1.5, cex.axis=1.5)
summary(resids)

#===Parametric modelling===#
#===hazard function, Fig
trans_haz <- pehaz(imputed$Grftsurvtime,imputed$Graftstatus,width=1, max.time=20)
haz_smooth <-muhaz(imputed$Grftsurvtime, imputed$Graftstatus, bw.smooth=20,
b.cor = "left", max.time =20)
plot(trans_haz,col="blue", lwd=2, ylab = "Estimated hazard function",
xlab="Years after transplant",cex.lab=1.5, cex.axis =1.5)
lines(haz_smooth, lwd=2, col="red")

#=== AFT models
wei <- survreg(for_sur~donor_type+delayed_gf+recip_age3+diabetes+ethnicity
+new_gender,dist="weibull",data=imputed)
logn <- survreg(for_sur~donor_type+delayed_gf+recip_age3+diabetes+ethnicity
+new_gender,dist="lognormal",data=imputed)
loglog <- survreg(for_sur~donor_type+delayed_gf+recip_age3+diabetes+ethnicity+
new_gender,dist="loglogistic",data=imputed)
summary()
AIC(wei)
AIC(logn)
AIC(loglog)

#Dev resid for weibull model fit, Fig
dev_wei<-residuals(wei,type="deviance")
plot(dev_wei,col="red",ylab="Deviance residuals Weibull model",cex.lab=1.5,
cex.axis =1.5)
abline(h=0, lty=2)
```

```
#===Alternatives variable selection
null<-coxph(imputed$Grftsurvtime,imputed$Graftstatus)~1
full<-coxph(surv20~dnr_age+donor_type+renal_disease+hypertension+urological+
inherited+no_complication+delayed_gf+
AR_clinical+AR_histological+new_gender+new_bloodgroup+ethnicity+recip_age
+diabetes,data = imputed_20yr)
cox_17<-step(full,scope = list(lower = null), direction="backward")#doforforward
summary(cox_17)
summary(cox_17)
cox.zph(cox_17)

regfit.full<-regsubsets(for_sur~dnr_age+donor_type+renal_disease+hypertension
+urological+inherited+no_complication+delayed_gf+
AR_clinical+AR_histological+new_gender+new_bloodgroup+ethnicity+recip_age+
diabetes, data = imputed)
reg.summary<-summary(regfit.full)
which.min(reg.summary$cp)
coef(regfit.full,scale="Cp",7)
```
