# Supplementary material for: Modeling Long-Term Graft Survival With Time-Varying Covariate Effects: An Application to a Single Kidney Transplant Centre in Johannesburg, South Africa
Source: Front Public Health. 2019 Jul 25;7:201. doi: 10.3389/fpubh.2019.00201 (PMC6669915; doi:10.3389/fpubh.2019.00201)
Supplement: Supplementary file 2 [file Data_Sheet_2.docx]

\documentclass[12pt,onesided,a4paper]{article}

\setlength{\textwidth}{17.0cm}

\setlength{\oddsidemargin}{-0.1cm}

\setlength{\textheight}{25.0cm}

\setlength{\topmargin}{-0.2in}

\renewcommand{\baselinestretch}{1.2}

\usepackage{latexsym}

\usepackage{amssymb}

\usepackage{pifont}

\usepackage{graphicx}

\usepackage{float}

\usepackage{amsmath}

\usepackage{amsthm}

\usepackage{fancyhdr}

\usepackage[none]{hyphenat}

\usepackage{setspace}

\usepackage[toc,page]{appendix}

%\usepackage{natbib}% biblatex can also be used with different citation commands

\usepackage[numbers,sort&compress]{natbib}%compress gives e.g.12-14 in reference

%\usepackage[numbers,sort]{natbib}

\usepackage{url}

%\usepackage[utf8]{inputenc}

\bibliographystyle{unsrtnat}

%\bibliographystyle{plainnat}

%\bibliography{test}

\usepackage[english]{babel}

\usepackage[section]{placeins}

\usepackage{titlesec}

\usepackage[colorlinks=true,linkcolor=blue,citecolor=red]{hyperref}

\usepackage[labelfont=bf]{caption}%\rhead{Share\LaTeX}

%\rhead{Share\LaTeX}

%\lhead{Guides and tutorials}

\usepackage[nottoc,notlof,notlot]{tocbibind}

\newcommand{\bbeta}{\mbox{\boldmath $\beta$}}

\newcommand{\balpha}{\mbox{\boldmath $\alpha$}}

\newcommand{\bX}{\mbox{\boldmath $X$}}

%\newcommand{\bx}{\mbox{\boldmath $x$}}

\newcommand{\br}{\mbox{\boldmath $r$}}

\newcommand{\tran}{'}

\DeclareMathOperator*{\argmin}{arg\,min}

\DeclareMathOperator*{\argmax}{arg\,max}

\usepackage{caption}

\usepackage{upgreek}

\usepackage{threeparttable}

\usepackage{fixltx2e}

%\captionsetup[table]{skip=5pt}

\newcommand{\xmark}{\ding{55}}

\usepackage{xr-hyper}

\usepackage{hyperref}

\newcommand{\beginsupplement}{%

\setcounter{table}{0}

\renewcommand{\thetable}{S\arabic{table}}%

\setcounter{figure}{0}

\renewcommand{\thefigure}{S\arabic{figure}}%

}

\externaldocument[A-]{Revised_manuscript_ed2}[Revised_manuscript_ed2.pdf]% <- full or relative path

\begin{document}

\onecolumn

\title{Supplementary Material - long-term graft survival with time-varying covariate effects: An application to a single kidney transplant centre in Johannesburg, South Africa}

\maketitle

\maketitle

\beginsupplement

\section{Supplementary Tables and Figures}

\begin{figure}[h]

\centering

\includegraphics[height=10.5cm]{paperFig/Fig9.jpg}

\caption{Kaplan-Meier estimates of a kernel-smoothed hazard function}

\label{hazP}

\end{figure}

\pagebreak

% Table generated by Excel2LaTeX from sheet 'Table 3'

\begin{table}[htbp]

\centering

\caption{Summary of the four models described in Table 2}

\begin{tabular}{lrcr}

\hline

\textbf{Model} & \multicolumn{1}{l}{\textbf{Variables}} & \textbf{HR(95\% CI)} & \textbf{\textit{p}-value} \\

\hline

& \multicolumn{1}{l}{Donor type} & 0.68(0.46-0.99) & 0.045 \\

& \multicolumn{1}{l}{Renal ESKD} & 0.80(0.59-1.08) & 0.140 \\

& \multicolumn{1}{l}{Hypertension ESKD} & 0.83(0.61-1.12) & 0.220 \\

& \multicolumn{1}{l}{Urological ESKD} & 0.76(0.48-1.21) & 0.249 \\

& \multicolumn{1}{l}{Inherited ESKD} & 0.65(0.42-0.99) & 0.047 \\

\textbf{1} & \multicolumn{1}{l}{Surgical complication} & 0.86(0.69-1.06) & 0.152 \\

& \multicolumn{1}{l}{Delayed graft function} & 1.39(1.11-1.73) & 0.003 \\

& \multicolumn{1}{l}{Diabetes at transplant} & 1.57(1.09-2.26) & 0.017 \\

& \multicolumn{1}{l}{Recipient ethnicity} & 1.44(1.12-1.86) & 0.004 \\

& \multicolumn{1}{l}{Donor age} & 1.00(0.99-1.01) & 0.654 \\

& \multicolumn{1}{l}{Recipient age} & 1.03(1.02-1.04) & $<$0.001 \\

& \multicolumn{1}{l}{Histological acute rejection} & 1.17(0.85-1.60) & 0.330 \\

\hline

& \multicolumn{1}{l}{Donor type} & 0.66(0.46-0.95) & 0.026 \\

& \multicolumn{1}{l}{Inherited ESKD} & 0.78(0.54-1.13) & 0.182 \\

& \multicolumn{1}{l}{Surgical complication} & 0.86(0.69-1.06) & 0.160 \\

\textbf{2} & \multicolumn{1}{l}{Delayed graft function} & 1.41(1.14-1.75) & 0.002 \\

& \multicolumn{1}{l}{Diabetes at transplant} & 1.53(1.07-2.18) & 0.018 \\

& \multicolumn{1}{l}{Recipient ethnicity} & 1.45(1.17-1.78) & 0.001 \\

& \multicolumn{1}{l}{Recipient age} & 1.03(1.02-1.04) & $<$0.001 \\

\hline

& \multicolumn{1}{l}{Donor type} & 0.66(0.45-0.95) & 0.025 \\

& \multicolumn{1}{l}{Delayed graft function} & 1.46(1.18-1.81) & $<$0.001 \\

\textbf{3} & \multicolumn{1}{l}{Diabetes at transplant} & 1.57(1.10-2.23) & 0.012 \\

& \multicolumn{1}{l}{Recipient ethnicity} & 1.49(1.21-1.83) & $<$0.001 \\

& \multicolumn{1}{l}{Recipient age} & 1.03(1.02-1.04) & $<$0.001 \\

\hline

& \multicolumn{1}{l}{Donor type} & 0.62(0.43-0.90) & 0.012 \\

& \multicolumn{1}{l}{Delayed graft function} & 1.49(1.21-1.85) & $<$0.001 \\

& \multicolumn{1}{l}{Diabetes at transplant} & 1.59(1.12-2.28) & 0.010 \\

\textbf{4} & \multicolumn{1}{l}{Recipient ethnicity} & 1.51(1.22-1.85) & 0.000 \\

& \multicolumn{1}{l}{Recipient age} & 1.03(1.02-1.04) & $<$0.001 \\

& \multicolumn{1}{l}{Donor-recipient gender \textit{(f-f)}} & 1.48(1.09-2.02) & 0.013 \\

& \textit{(f-m)} & 1.25(0.97-1.60) & 0.082 \\

& \textit{(m-f)} & 1.16(0.87-1.55) & 0.320 \\

\hline

\end{tabular}%

\label{4models}%

\end{table}%

\pagebreak

\begin{figure}[h]

\centering

\includegraphics[height=14.5cm]{paperFig/Fig11.jpg}

\caption{Plot of graft survival probability vs. years post transplant for the categorical variables in Model 4}

\label{KM}

\end{figure}

\pagebreak

\begin{figure}[h]

\centering

\includegraphics[height=20.5cm]{paperFig/Fig4.jpg}

\caption{Assessing of PH assumption: graphs of the scaled Schoenfeld residuals versus transformed time for each covariate in the Cox PH model. The solid and the broken lines represent the smoothing spline fit and the $\pm$2 standard error for the fit.}

\label{SCRT}

\pagebreak

\end{figure}

\begin{figure}[h]

\centering

\includegraphics[height=20.5cm]{paperFig/Fig5.jpg}

\caption{Assessing of PH assumption:graphs of observed test processes with 50 simulated processes for each covariate in the Cox PH model. The solid black profile signifies the observed pattern.}

\label{OTP}

\end{figure}

\pagebreak

% Table generated by Excel2LaTeX from sheet 'Table 6'

\begin{table}[htbp]

\centering

\caption{Result of the extended Cox PH model with main effects of the covariates with time-varying effects and their interaction with time}

\begin{tabular}{lrr}

\hline

\textbf{Variable} & \textbf{HR} & \textbf{\textit{p}-value} \\

\hline

Donor type & 0.63 & 0.017 \\

Delayed graft function & 1.73 & $<$0.001 \\

Diabetes at transplant & 1.18 & 0.537 \\

Donor-recipient gender & & \\

\multicolumn{1}{c}{\textit{f-f}} & 1.47 & 0.016 \\

\multicolumn{1}{c}{\textit{f-m}} & 1.22 & 0.120 \\

\multicolumn{1}{c}{\textit{m-f}} & 1.14 & 0.371 \\

Recipient ethnicity & 1.84 & $<$0.001 \\

Recipient age & 1.03 & $<$0.001 \\

(Delayed graft function) $\times$ time & 1.04 & 0.131 \\

(Diabetes at transplant) $\times$ time & 0.95 & 0.113 \\

(Recipient ethnicity) $\times$ time & 1.05 & 0.036 \\

\hline

\textbf{Proportionality test} & & \textbf{0.083} \\

\hline

\end{tabular}%

\label{tint}%

\end{table}%

\pagebreak

% Table generated by Excel2LaTeX from sheet 'Table A1'

\begin{table}[htbp]

\footnotesize

\centering

\caption{Comparing variables selected with the purposeful method of variable selection and the automated methods}

\begin{tabular}{lccccc}

\hline

\textbf{Variables} & \textbf{Purposeful} & \textbf{Stepwise} & \textbf{Backwards} & \textbf{Forward} & \textbf{Best subset} \\

\hline

Recipient age & \checkmark & \checkmark & \checkmark & \checkmark & \checkmark\\

Donor type & \checkmark & \checkmark & \checkmark & \checkmark & \checkmark\\

Recipient ethnicity & \checkmark & \checkmark & \checkmark & \checkmark & \checkmark\\

Diabetes at transplant& \checkmark & \checkmark & \checkmark & \checkmark & \checkmark\\

Donor-recipient gender & \checkmark & \checkmark & \checkmark & \checkmark & \checkmark\\

Delayed graft function & \checkmark & \checkmark & \checkmark & \checkmark & \checkmark\\

Inherited ESKD & \xmark&\checkmark & \checkmark & \checkmark & \checkmark \\

Surgical complication & \xmark & \xmark & \xmark & \xmark & \checkmark \\

\hline

\end{tabular}%

\label{varse}%

\end{table}%

\section{R codes}

\begin{verbatim}

#R codes for analyses

#===reading in the libraries

rm(list=ls())

library(survival)

library(gof)

library(BaylorEdPsych)

library(dplyr)

library(missForest)

library(survminer)

library(muhaz)

library(MASS)

library(bshazard)

library(ISLR)

library(leaps)

library(timereg)

library(flexsurv)

#===importing data from a csv file

CYA <- read.csv("C:/Users/Ike/Desktop/MSc_data/CYA.csv", header =TRUE)

str(CYA)

summary(CYA)

#===Data manipulation after several steps of data cleaning

CYA2 <- CYA

CYA2$ethnicity<-factor(CYA2$ethnicity)

CYA2$new_bloodgroup<-factor(CYA2$new_bloodgroup)

CYA2$new_gender<-factor(CYA2$new_gender)

CYA2$ethnicity <- relevel(CYA2$ethnicity, ref="white")

CYA2$new_gender <- relevel(CYA2$new_gender, ref="male-male")

CYA2$diabetes<-CYA2$diabetes_tx

CYA2$recip_age<-CYA2$Recip_Age_cont

CYA2 <- CYA2[c(-3,-9,-10,-11,-12,-15)]

names(CYA2)

#===arrange variables

CYA2<-CYA2 %>%dplyr:: select(job_id,Grftsurvtime, Graftstatus,recip_age,

renal_disease,hypertension, urological,inherited, AR_clinical,

AR_histological,everything())

#===Missing completely at random test

Mcar<-LittleMCAR(data.frame(CYA2[4:18]))

Mcar[c("chi.square", "df", "p.value")]

#===subset data to 20 years because of few survival recorded

towards the end of the study

CYA_20<-CYA2

CYA_20$Grftsurvtime[CYA2$Grftsurvtime>20]<-20

CYA_20$Graftstatus[CYA2$Grftsurvtime>20]<-0

surv20<-with(CYA_20, Surv(Grftsurvtime,Graftstatus))

summary(CYA2$Grftsurvtime)

surv_rate<-survfit(Surv(Grftsurvtime, Graftstatus)~ 1, data=CYA_20)

summary(surv_rate, times=c(1,5,10,15,20))

#===Extract dates of transplant & survival variables, Fig2A

timeD<-read.csv("C:/Users/Ike/Desktop/timeseries.csv", header = TRUE)

time_series<-subset(timeD,timeD$Transplant_era=="CYA")

time_series2<-as.Date(time_series$date_tx,"%m/%d/%Y")

class(time_series2)

time_series3<-format(time_series2, format="%Y")

time_series_F<-cbind(time_series,time_series3)

time_series_T<-table(time_series_F$time_series3)

time_series_D<-data.frame(time_series_T)

head(time_series_D)

barplot(Freq~Var1, data = time_series_D,cex.lab=1.5,cex.axis=1.5,

xlab = "Years of transplant",ylab = "Frequency",cex.names=1.5)

#===Histogram and survival curve, Fig. 2B and C

trans_km <-survfit(Surv(CYA_20$Grftsurvtime, CYA_20$Graftstatus)~1)

trans_km

plot(trans_km)

summary(trans_km,times=1)

par(mfrow=c(2,2))

his_pre<-hist(CYA_20$Grftsurvtime,xlab = "Years after transplant",

main = NULL, col="gray", ylim=c(0,400),cex.lab=1.5, cex.axis =1.5)

plot(trans_km,xlab="Years after transplant", ylab= "Survival

probability", mark.time = T, col="black", conf.int =F,cex.lab=1.5, cex.axis =1.5)

#===Hazard rate estimates,Fig 2D

uni<- bshazard(Surv(Grftsurvtime,Graftstatus)~1, CYA_20)

plot(uni,col="blue", lwd=2, ylab = "Estimated hazard function",

xlab="Years after transplant",

cex.lab=1.5, cex.axis =1.5)

#plot(uni)

plot(uni$time,uni$hazard*1000,col="black", type = "l", lwd = 3,

ylab = "Graft failure rate per 1000 PY", xlab="Years after transplant",

cex.lab=1.5, cex.axis =1.5)

#===Imputation

set.seed(81)

for_imp <- missForest(mspap, verbose = TRUE,maxiter = 5, ntree = 500)

for_imp$OOBerror

imputed<-for_imp$ximp

is.na(imputed)

imputed<-imputed %>%dplyr:: select(job_id,Grftsurvtime,Graftstatus,

recip_age, dnr_age, everything())

names(imputed)

#===univariate analysis

uni_all<-colnames(imputed)[4:18]

for (i in 1:length(uni_all)){

print(uni_all[i])

print(coxph(Surv(Grftsurvtime,Graftstatus) ~ get(uni_all[i]), data=imputed))

}

for_sur<-with(imputed, Surv(Grftsurvtime,Graftstatus))

#===centering

imputed$recip_age3<- scale(imputed$recip_age, center = TRUE,scale = FALSE)

imputed$dnr_age3<- scale(imputed$dnr_age, center = TRUE,scale = FALSE)

#===purposeful method of variable selection===#

purv2<-coxph(for_sur~donor_type+renal_disease+hypertension+

urological+inherited+no_complication+

delayed_gf+diabetes+ethnicity+dnr_age3+recip_age3+AR_histological,

data=imputed)

summary(purv2)

-2*purv2$loglik[2]

#===drop dnrage

purv3<-coxph(for_sur~donor_type+renal_disease+hypertension+urological

+inherited+no_complication+

delayed_gf+diabetes+ethnicity+recip_age3+AR_histological,data=imputed)

summary(purv3)

-2*purv3$loglik[2]

# likelihood ratio test:

X.lr3=-2*purv3$loglik[2]-(-2*purv2$loglik[2]) # test statistics

X.lr3

1-pchisq(X.lr3,1) # p-value

delta.coff<-abs((coef(purv3)-coef(purv2)[-10])/coef(purv2)[-10])

round(delta.coff,5)

#===drop AR_histological

purv4<-coxph(for_sur~donor_type+renal_disease+hypertension+urological

+inherited+no_complication+

delayed_gf+diabetes+ethnicity+recip_age3,data=imputed)

summary(purv4)

-2*purv4$loglik[2]

# likelihood ratio test:

X.lr4=-2*purv4$loglik[2]-(-2*purv3$loglik[2]) # test statistics

X.lr4

1-pchisq(X.lr4,1) # p-value

delta.coff<-abs((coef(purv4)-coef(purv3)[-11])/coef(purv3)[-11])

round(delta.coff,5)

#===urological,renal_d and hyper were dropped because of urological influence

purv5<-coxph(for_sur~donor_type+renal_disease+hypertension+inherited

+no_complication+

delayed_gf+diabetes+ethnicity+recip_age3,data=imputed)

summary(purv5)

-2*purv5$loglik[2]

X.lr5=-2*purv5$loglik[2]-(-2*purv4$loglik[2])

X.lr5

1-pchisq(X.lr5,1) # p-value

delta.coff<-abs((coef(purv5)-coef(purv4)[-4])/coef(purv4)[-4])

round(delta.coff,5)

#===restart the model building process

purv6<-coxph(for_sur~donor_type+inherited+no_complication+

delayed_gf+diabetes+ethnicity+recip_age3,data=imputed)

summary(purv6)

-2*purv6$loglik[2]

#===inherited

purv7<-coxph(for_sur~donor_type+no_complication+

delayed_gf+diabetes+ethnicity+recip_age3,data=imputed)

summary(purv7)

-2*purv7$loglik[2]

# likelihood ratio test:

X.lr7=-2*purv7$loglik[2]-(-2*purv6$loglik[2]) # test statistics

X.lr7

1-pchisq(X.lr7,1) # p-value

delta.coff7<-abs((coef(purv7)-coef(purv6)[-2])/coef(purv6)[-2])

round(delta.coff7,5)

#===nocomplication

purv7a<-coxph(for_sur~donor_type+

delayed_gf+diabetes+ethnicity+recip_age3,data=imputed)

summary(purv7a)

-2*purv7a$loglik[2]

# likelihood ratio test:

X.lr7a=-2*purv7a$loglik[2]-(-2*purv7$loglik[2]) # test statistics

X.lr7a

1-pchisq(X.lr7a,1) # p-value

delta.coff7<-abs((coef(purv7a)-coef(purv7)[-2])/coef(purv7)[-2])

round(delta.coff7,5)

#===Add variables not sig at 25%===#

#===gender

purv8<-coxph(for_sur~donor_type+

delayed_gf+diabetes+ethnicity+recip_age3+new_gender,data=imputed)

summary(purv8)

-2*purv8$loglik[2]

# likelihood ratio test:

X.lr8=-2*purv7a$loglik[2]-(-2*purv8$loglik[2]) # test statistics

X.lr8

1-pchisq(X.lr8,1) # p-value

#delta.coff8<-abs((coef(purv7)-coef(purv8)[-7])/coef(purv8)[-7])

#round(delta.coff8,5)

#===AR_histological, bloodgroup

purv9<-coxph(for_sur~donor_type+

delayed_gf+diabetes+ethnicity+recip_age3+new_gender+new_bloodgroup,

data=imputed)

summary(purv9)

-2*purv9$loglik[2]

X.lr9=-2*purv8$loglik[2]-(-2*purv9$loglik[2]) # test statistics

X.lr9

1-pchisq(X.lr9,1) # p-value

delta.coff9<-abs((coef(purv8)-coef(purv9)[-7])/coef(purv9)[-7])

round(delta.coff9,5)

#===Final model====#

Finover<-coxph(for_sur~donor_type+

delayed_gf+recip_age3+diabetes+new_gender+ethnicity,data=imputed)

summary(Finover)

vif(Finover)

#===Linearity assessment

smooth_SEcurve<-function(yy,xx){

list_x<-min(xx) + ((0:100)/100)*(max(xx)-min(xx))

yy_xx<-predict(loess(yy~xx),se=T,newdata=data.frame(xx=list_x))

lines(yy_xx$fit ~list_x, lwd=2,col="blue")

lines(yy_xx$fit - qt(0.975,yy_xx$df)*yy_xx$se.fit~list_x, lty=2)

lines(yy_xx$fit + qt(0.975,yy_xx$df)*yy_xx$se.fit~list_x, lty=2)

}

mart <-coxph(Surv(imputed$Grftsurvtime, imputed$Graftstatus)~1)

martR <-residuals(mart, type="martingale")

par(mfrow=c(2,2))

plot(martR~imputed$recip_age,xlab="recip_age", ylab = "Martingale

residual", col="red", cex.lab=1.5, cex.axis =1.5)

smooth_SEcurve(martR,imputed$recip_age)

#pspline

ps_cox <-coxph(for_sur~donor_type+delayed_gf+diabetes+ethnicity+pspline

(recip_age3,df=4)

+new_gender,data=imputed)

ps_cox

termplot(ps_cox,se=T, terms = 1,ylabs = "Log hazard", col.term = "blue",

col.se = "blue",cex.lab=1.5,

cex.axis =1.5,lwd.term = 2,lwd.se = 2)

#===PH assessment

cox.zph(Finover)

par(mfrow=c(2,2))

plot(cox.zph(Finover),ann=T, var=1,col="blue",lwd=2)

abline(h=0, lty=2, col="red")

plot( cox.zph(Finover), ann=T, var=2, col="blue",lwd=2 )

abline(h=0, lty=2, col="red")

plot( cox.zph(Finover), ann=T, var=3 , col="blue",lwd=2)

abline(h=0, lty=2, col="red")

plot( cox.zph(Finover), ann=T, var=4, col="blue",lwd=2)

abline(h=0, lty=2, col="red")

plot( cox.zph(Finover), ann=T, var=5, col="blue",lwd=2)

abline(h=0, lty=2, col="red")

plot( cox.zph(Finover), ann=T, var=6,col="blue", lwd=2)

abline(h=0, lty=2, col="red")

plot( cox.zph(Finover), ann=T, var=7,col="blue", lwd=2)

abline(h=0, lty=2, col="red")

plot( cox.zph(Finover), ann=T, var=8,col="blue", lwd=2)

cmr <- cumres(Finover,R=50)

cmr

par(mfrow=c(2,2))

plot(cmr,legend = c("type2"))

abline(h=0, lty=2, col="red")

par(mfrow=c(2,2))

KM1= survfit(Surv(Grftsurvtime, Graftstatus)~ donor_type, data=imputed)

plot(KM1,lwd =2, xlab="Years after transplant",ylab= "Survival

probability", col=c("red","black"), conf.int = F,cex.lab=1.5, cex.axis =1.5)

legend("topright",c("cadaveric","living"),col=c("red","black"),lty=1,bty='n',

title = "Donor type",text.font = 3,cex = 1.5)

KM2 = survfit(Surv(Grftsurvtime, Graftstatus)~ ethnicity, data=imputed)

plot(KM2,lwd =2, xlab="Years after transplant",ylab= "Survival probability",

col=c("red","black"), conf.int = F,cex.lab=1.5, cex.axis =1.5)

legend("topright",c("white", "non-white"),col=c("red","black"),lty=1,bty='n',

title = "Recipient ethnicity",text.font = 3,cex = 1.5)

KM3 = survfit(Surv(Grftsurvtime, Graftstatus)~ delayed_gf, data=imputed)

plot(KM3,lwd =2, xlab="Years after transplant",ylab= "Survival probability",

col=1:2, conf.int = F,cex.lab=1.5, cex.axis =1.5)

legend("topright",c("no","yes"),col=1:2,lty=1,bty='n',title = "Delayed graft

function",text.font = 3,cex = 1.5)

KM4= survfit(Surv(Grftsurvtime, Graftstatus)~ diabetes, data=imputed)

plot(KM4,lwd =2, xlab="Years after transplant",ylab= "Survival probability",

col=1:2, conf.int = F,cex.lab=1.5, cex.axis =1.5)

legend("topright",c("no","yes"),col=1:2,lty=1,bty='n',title = "Diabetes at

transplant",text.font = 3,cex = 1.5)

KM4= survfit(Surv(Grftsurvtime, Graftstatus)~ new_gender, data=imputed)

plot(KM4,lwd =2, xlab="Years after transplant",ylab= "Survival probability",

col=1:4, conf.int = F,cex.lab=1.5, cex.axis =1.5)

legend("topright",c("f-m","f-f","m-f","m-m"),col=1:4,lty=1,bty='n',title =

"Donor-recipient gender",

text.font = 3,cex = 1.5)

#===overall Cox model fit

coxsnellres=imputed$Graftstatus-resid(Finover, type="martingale")

fitres=survfit(coxph(Surv(coxsnellres, imputed$Graftstatus)~1, method

= 'breslow'),type='aalen')

fitres

plot(fitres$time, -log(fitres$surv), type = 'p', xlab = ' Cox-snell residuals',

ylab ='Estimated cumulative hazard function',lwd=2.5,lty=6,cex.lab=1.5,

cex.axis =1.5)

abline(0,1, col='red', lwd=2,lty=1)

par(mfrow=c(2,2))

dev_cox<-residuals(Finover,type="deviance")

plot(dev_cox,col="red",ylab="Deviance residual for Cox PH model",cex.lab=1.5,

cex.axis =1.5)

abline(h=0, lty=2)

identify(dev_cox)

#===Additive hazard===

Finover4<-aalen(for_sur~donor_type+delayed_gf+diabetes+ethnicity+recip_age

+new_gender,max.time = 20, residuals = 1,data=imputed)

Finover5<-aalen(for_sur~donor_type+const(delayed_gf)+const(diabetes)+ethnicity+

const(recip_age)+const(new_gender),residuals=1,data=imputed)

Finover4

Finover5

par(mfrow=c(2,2))

plot(Finover4,cex.lab=1.5, cex.axis =1.5,lwd=2)

#==cumulative martingal residual

par(mfrow=c(2,2))

X<-model.matrix(~donor_type+delayed_gf+diabetes+ethnicity+recip_age3+

new_gender,data=imputed,residuals=1,n.sim=100)

resids<-cum.residuals(Finover4,cum.resid=1,modelmatrix=X,n.sim=100,data=imputed)

plot(resids,score=1,cex.lab=1.5, cex.axis=1.5)

summary(resids)

#===Parametric modelling===#

#===hazard function, Fig

trans_haz <- pehaz(imputed$Grftsurvtime,imputed$Graftstatus,width=1, max.time=20)

haz_smooth <-muhaz(imputed$Grftsurvtime, imputed$Graftstatus, bw.smooth=20,

b.cor = "left", max.time =20)

plot(trans_haz,col="blue", lwd=2, ylab = "Estimated hazard function",

xlab="Years after transplant",cex.lab=1.5, cex.axis =1.5)

lines(haz_smooth, lwd=2, col="red")

#=== AFT models

wei <- survreg(for_sur~donor_type+delayed_gf+recip_age3+diabetes+ethnicity

+new_gender,dist="weibull",data=imputed)

logn <- survreg(for_sur~donor_type+delayed_gf+recip_age3+diabetes+ethnicity

+new_gender,dist="lognormal",data=imputed)

loglog <- survreg(for_sur~donor_type+delayed_gf+recip_age3+diabetes+ethnicity+

new_gender,dist="loglogistic",data=imputed)

summary()

AIC(wei)

AIC(logn)

AIC(loglog)

#Dev resid for weibull model fit, Fig

dev_wei<-residuals(wei,type="deviance")

plot(dev_wei,col="red",ylab="Deviance residuals Weibull model",cex.lab=1.5,

cex.axis =1.5)

abline(h=0, lty=2)

#===Alternatives variable selection

null<-coxph(imputed$Grftsurvtime,imputed$Graftstatus)~1

full<-coxph(surv20~dnr_age+donor_type+renal_disease+hypertension+urological+

inherited+no_complication+delayed_gf+

AR_clinical+AR_histological+new_gender+new_bloodgroup+ethnicity+recip_age

+diabetes,data = imputed_20yr)

cox_17<-step(full,scope = list(lower = null), direction="backward")#doforforward

summary(cox_17)

summary(cox_17)

cox.zph(cox_17)

regfit.full<-regsubsets(for_sur~dnr_age+donor_type+renal_disease+hypertension

+urological+inherited+no_complication+delayed_gf+

AR_clinical+AR_histological+new_gender+new_bloodgroup+ethnicity+recip_age+

diabetes, data = imputed)

reg.summary<-summary(regfit.full)

which.min(reg.summary$cp)

coef(regfit.full,scale="Cp",7)

\end{verbatim}

\end{document}
