# Supplementary figures and images for: Modeling Long-Term Graft Survival With Time-Varying Covariate Effects: An Application to a Single Kidney Transplant Centre in Johannesburg, South Africa
Source: Front Public Health. 2019 Jul 25;7:201. doi: 10.3389/fpubh.2019.00201 (PMC6669915; doi:10.3389/fpubh.2019.00201)

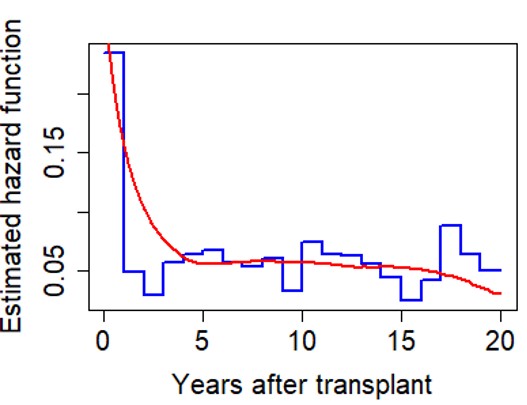

Supplement: Supplementary file 3 [file Image_1.JPEG]

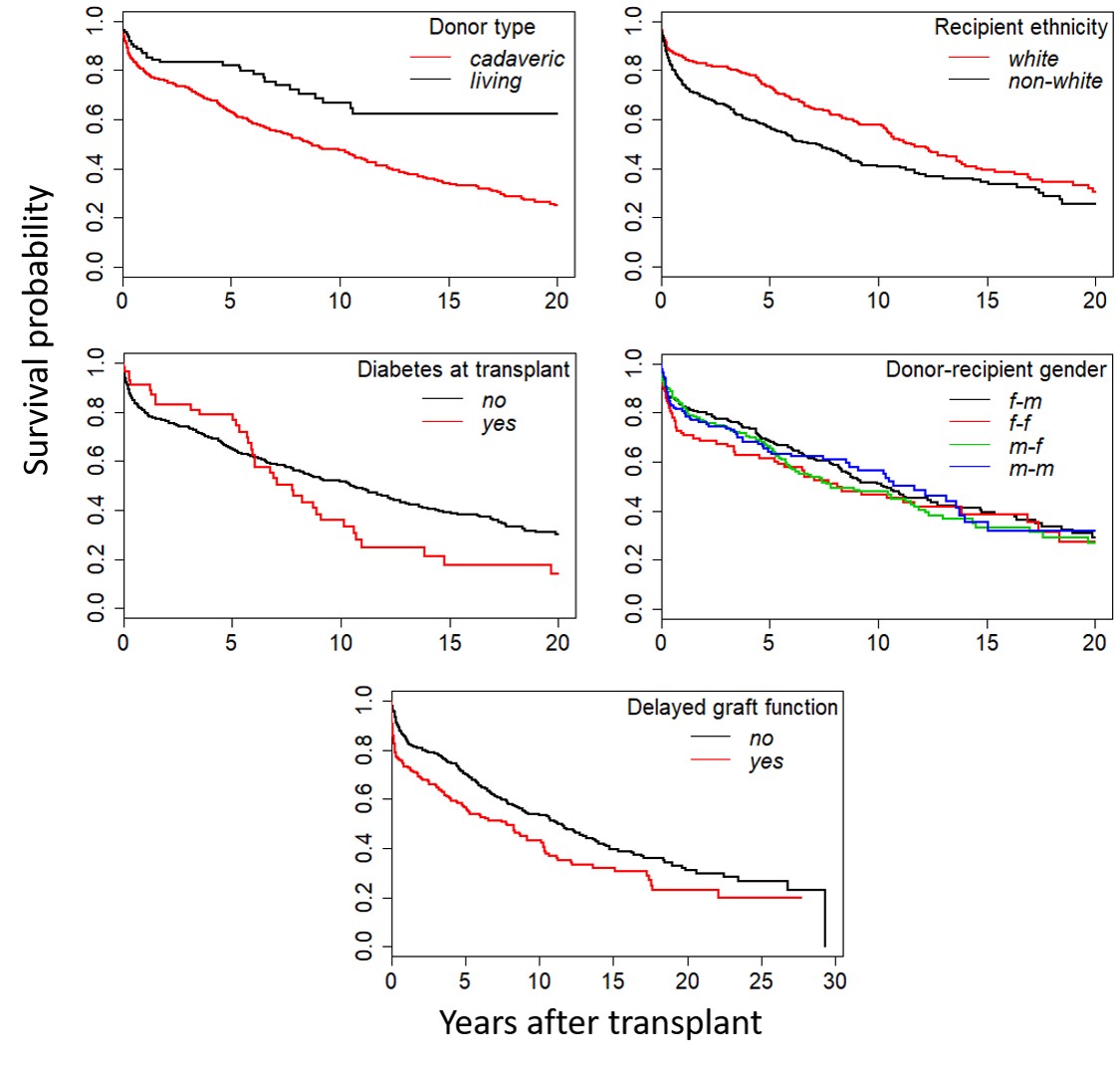

Supplement: Supplementary file 4 [file Image_2.JPEG]

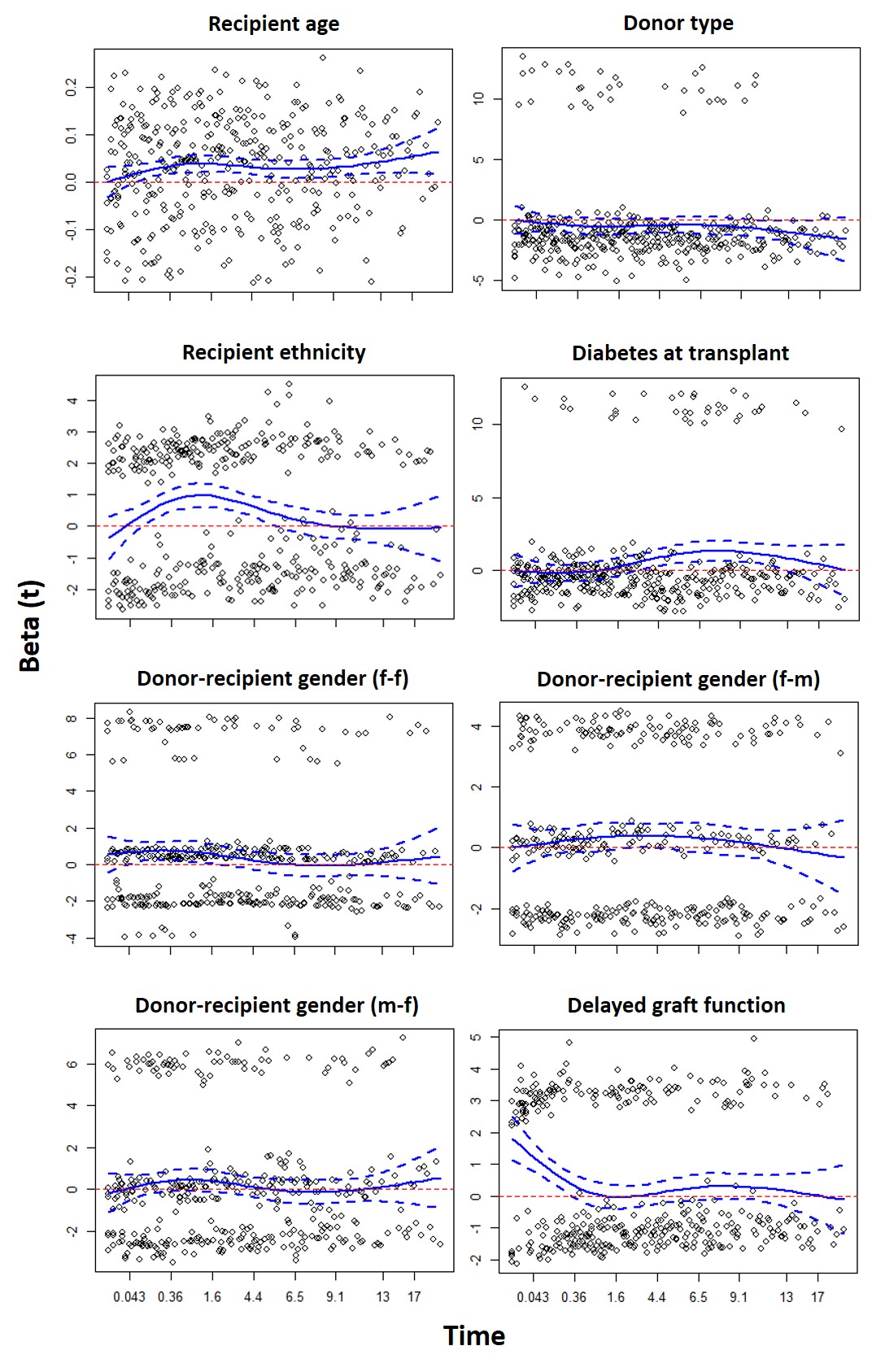

Supplement: Supplementary file 5 [file Image_3.JPEG]

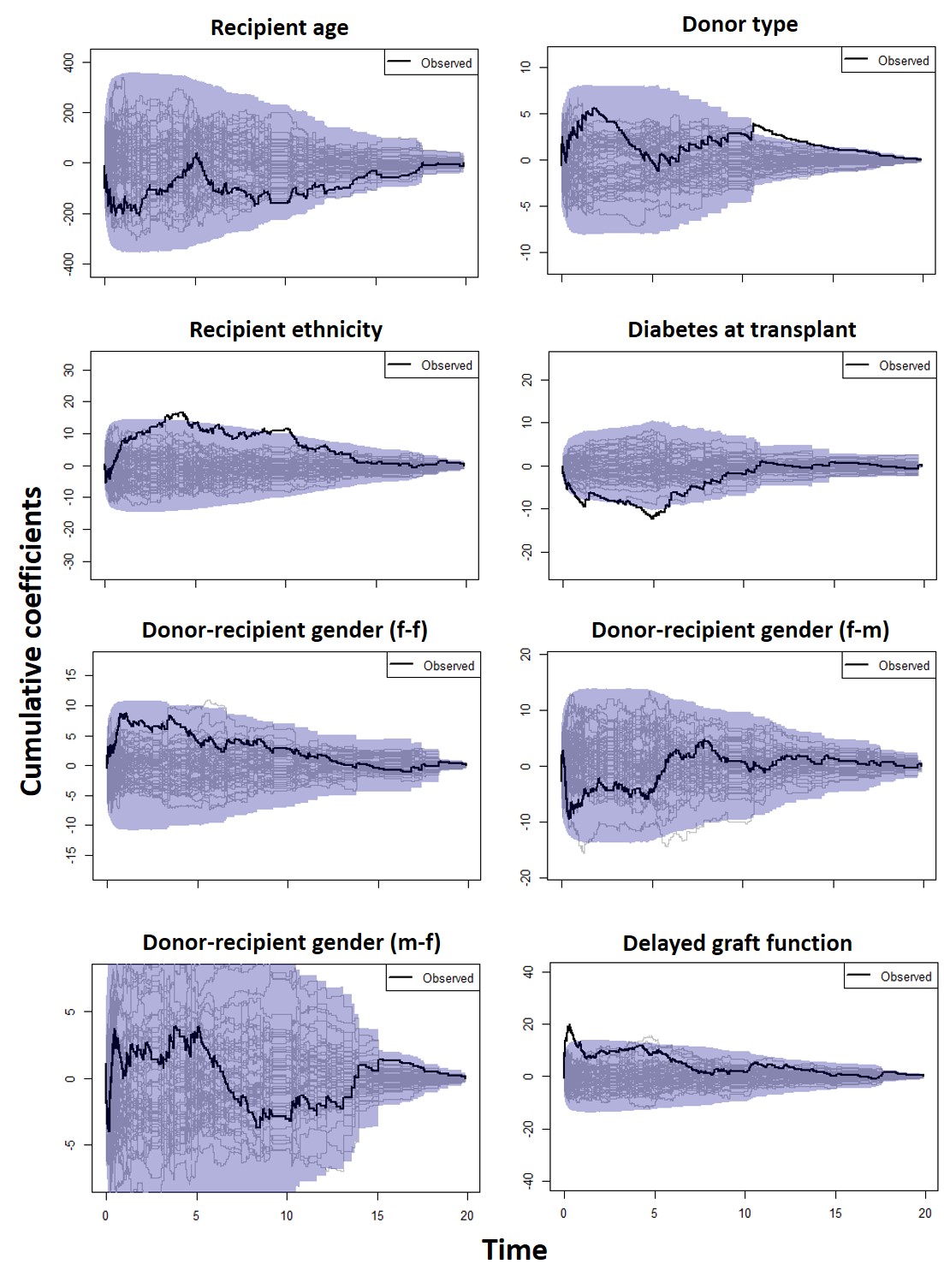

Supplement: Supplementary file 6 [file Image_4.JPEG]
